# Optimising a digital parent-based intervention to improve adolescent health risk behaviours in families living in socio-economically disadvantaged communities: findings from the *Health4Life Parents & Teens* factorial randomised trial

**DOI:** 10.1016/j.lanwpc.2026.101987

**Published:** 2026-09-23

**Authors:** Katrina E. Champion, Lily Davidson, Matthew Sunderland, Emily Hunter, Bonnie Spring, Louise Thornton, Alex Haidinger, Tara Finn, Bridie Osman, Cath Chapman, Tracy Burrows, Tim Slade, Stephanie R. Partridge, Lauren A. Gardner, Belinda J. Parmenter, Louise A. Baur, Maree Teesson, Cathrine Mihalopoulos, George Johnson, Emily Stockings, Lyra Egan, Susan Woolfenden, Frances Kay, David R. Lubans, Nicola C. Newton

**Affiliations:** a The Matilda Centre for Research in Mental Health and Substance Use, University of Sydney, Sydney, NSW, Australia; b Sydney School of Public Health, The University of Sydney, Sydney, NSW, Australia; c Department of Preventive Medicine, Northwestern University Feinberg School of Medicine, Chicago, IL, USA; d Department of Behavioral Sciences and Social Medicine, Florida State University College of Medicine, Tallahassee, FL, USA; e School of Medicine and Public Health, The University of Newcastle, Callaghan, NSW, Australia; f Global Sport and Movement Collaborative, University of Newcastle, Callaghan, NSW, Australia; g Hunter Medical Research Institute, New Lambton Heights, NSW, Australia; h School of Health Sciences, College of Health, Medicine and Wellbeing, University of Newcastle, Callaghan, NSW, Australia; i Sydney Nursing School, The University of Sydney, Sydney, NSW, Australia; j Charles Perkins Centre, The University of Sydney, Sydney, NSW, Australia; k School of Health Sciences, University of New South Wales, Sydney, NSW, Australia; l School of Health, University of the Sunshine Coast, QLD, Australia; m Children's Hospital Westmead Clinical School, Sydney Medical School, The University of Sydney, Westmead, NSW, Australia; n School of Public Health and Preventive Medicine, Monash University, Australia; o Sydney Institute for Women, Children and their Families, Sydney Local Health District, Sydney, NSW, Australia; p Community Paediatric Research Group, Sydney Medical School, University of Sydney, Sydney, NSW, Australia; q Faculty of Sport and Health Sciences, University of Jyväskylä, Jyväskylä, Finland

**Keywords:** Adolescents, Parents, Digital intervention, Multiphase optimisation strategy

## Abstract

**Background:**

Adolescence is a critical period when health risk behaviours contributing to long-term disease burden emerge. Parent-based interventions show promise, yet engagement is often low and few exist for socioeconomically disadvantaged communities. We applied the Multiphase Optimisation Strategy (MOST) to identify a maximally effective, minimally burdensome digital intervention to support parents to improve adolescent health risk behaviours.

**Methods:**

A 2 × 2 × 2 × 2 factorial randomised trial was conducted with parents of adolescents (aged 11–15 years), living in a disadvantaged area of New South Wales, Australia (quintiles 1–2, Index of Relative Socio-economic Disadvantage). Participants were recruited via social media and in community settings and randomised to 1 of 16 experimental conditions via a Blockrand-generated scheme in R. All participants received access to six online modules targeting key health risk behaviours (core component). Four components were experimentally tested (present vs absent): text messages, tailored feedback, stress management training, and telehealth coaching, resulting in 16 possible intervention combinations. Participants and researchers were not masked to allocation. The primary outcome was 3-month change in parental encouragement of adolescent health habits (Health Habits subscale of Parenting to Reduce Adolescent Depression Anxiety Scale; possible range 0–12). Parent-reported perceived effort of completing each component was examined (5-point scale: ‘no effort at all’ to ‘huge effort’). The trial was prospectively registered (ACTRN12624001492549; 20/12/2024).

**Findings:**

Between April 23 and October 22 2025, we recruited and randomised 303 participants. One withdrew after randomisation, and ten were removed during analysis, resulting in 292 parents at baseline and 231 (79%) at follow-up. Tailored feedback (*B* = 0.21, p = 0.054, mean difference = 0.42, 95% CI = −0.01 to 0.85, Cohen's d = 0.26) and telehealth coaching (*B* = 0.31, p = 0.0051, mean difference = 0.62, 95% CI = 0.19 to 1.04, Cohen's d = 0.38) improved parental encouragement, while text messages had a negative effect (B = −0.33, p < 0.0026, mean difference = −0.66, 95% CI = −1.09 to −0.23, Cohen's d = −0.41). Telehealth coaching yielded the greatest improvements, but highest perceived effort (mean = 2.4/5, SD = 1.2). Tailored feedback achieved comparable gains, with significantly lower effort (mean = 2.1/5, SD = 1.0; p = 0.03). The optimised intervention comprised online modules and tailored feedback. No adverse events were reported.

**Interpretation:**

Using a highly efficient MOST factorial design, we identified a low-burden, highly scalable intervention for parents living in disadvantaged communities, ready for evaluation in a definitive randomised controlled trial.

**Funding:**

Medical Research Future Fund (2021535).

**Research in context**

**Evidence before this study**

Our systematic review of parent-based interventions targeting multiple adolescent health risk behaviours informed this study. MEDLINE, Embase, PsycInfo, Scopus, CINAHL, the Cochrane Database of Systematic Reviews and Cochrane Central Register of Controlled Trials were searched from 2010–May 2021. A list of search terms is provided in the Appendix. Eligible studies were randomised controlled trials (RCTs) of parent-based interventions addressing two or more of the following risk behaviours: alcohol use, smoking, poor diet, physical inactivity, sedentary behaviours, and poor sleep. Studies that directly targeted parents and assessed adolescent outcomes (11–18 years) were eligible. A total of 46 publications of 36 RCTs were identified. We systematically extracted the behaviour change techniques (BCTs) used in each intervention using a validated taxonomy. Parent-based interventions were associated with increased adolescent moderate-to-vigorous physical activity [OR = 1.82, 95% CI = 1.18 to 2.81], and reduced screen time (standardized mean difference [SMD] = −0.39, 95% CI = −0.62 to −0.16) and decreased discretionary food intake (SMD = −0.18; 95% CI = −0.30 to −0.06) compared to controls. However, there was some evidence that interventions increased the odds of ever using tobacco (OR = 1.47, 95% CI = 0.99 to 2.18) and of past month tobacco use (OR = 1.46, 95% CI = 1.12 to 1.90). Overall, effects were small, and the quality of evidence was moderate. Only one trial included parents experiencing socioeconomic disadvantage. The most effective BCTs were social support and feedback on parents' behaviour. The inclusion of youth-only components, in addition to parent components, appeared to be associated with beneficial program effects. We concluded that further research is needed to develop parent-based interventions to promote healthy sleep; improve efficacy, particularly for alcohol and tobacco use; and use novel experimental designs to identify optimal intervention component combinations to build increasingly efficient, effective and cost-effective interventions.

**Added value of this study**

This study was the first to use the highly efficient Multiphase Optimisation Strategy (MOST) framework to optimise a maximally effective and minimally burdensome digital intervention to support parents in promoting multiple health behaviours among adolescents. We conducted a factorial randomised trial among parents living in socio-economically disadvantaged communities across New South Wales, Australia to test the efficacy of four intervention components: text messages, tailored feedback, stress management training, and telehealth coaching (i.e., weekly phone calls from a trained health coach). Our findings showed that both tailored feedback and telehealth coaching significantly improved parental encouragement of adolescent health habits, however parents rated telehealth coaching as requiring more effort. Text messages had a negative effect on parental encouragement, while stress management had no effect. Considering both component efficacy and perceived effort for parents, the final intervention comprised the online learning modules and automated tailored feedback about their parenting practices.

**Implications of all the available evidence**

This study demonstrates the benefits of efficient intervention optimisation approaches using the MOST framework. Systematically testing intervention components individually, rather than evaluating multicomponent interventions as a whole package, provides crucial evidence on what components are essential and what can be eliminated to reduce burden and resource use. This study yielded a highly scalable, resource-efficient program, ready for evaluation in a full-scale randomised controlled trial alongside the existing online school-based Health4Life program among parent-adolescent dyads. These insights are relevant for developing and optimising other digital interventions, particularly in settings and populations where resources are constrained.

## Introduction

The second Lancet Commission on Adolescent Health and Wellbeing estimated that, by 2030, over one billion adolescents aged 10–24 years (around half of adolescents globally) will live in countries where preventable health problems threaten their wellbeing.^1^ Early and middle adolescence are critical developmental periods when health risk behaviours often emerge, including poor nutrition, inadequate physical activity, poor sleep, excessive recreational screen time, alcohol use and smoking/vaping (the “Big 6”).^2^ These behaviours commonly co-occur^2^ and track into adulthood, contributing to chronic disease and poor health outcomes across the life course.^3^ People who experience socio-economic disadvantage are disproportionately affected by the Big 6 due to structural and systemic barriers.^4^ In Australia, adolescents living in areas of greatest socioeconomic are more likely to drink alcohol at risky levels and to smoke, and less likely to meet fruit consumption guidelines, than those living in more advantaged areas.^5^ New approaches that address these interconnected factors and deliver effective interventions, particularly for adolescents experiencing disadvantage, are needed.

Parents, guardians and caregivers (herein referred to as parents) play a crucial role in shaping adolescent health through role modelling, rule-setting, monitoring, and communication.^6^^,^^7^ Existing parent interventions (i.e., programs delivered to parents to increase their knowledge, skills and confidence about parenting) have demonstrated efficacy in modifying adolescent health risk behaviours, especially when paired with a youth-based component.^8^ However, engaging parents is challenging, with research consistently highlighting a lack of time and competing demands as barriers to uptake and ongoing participation.^9^^,^^10^ This is especially relevant for parents experiencing socio-economic disadvantage, who can face additional challenges like financial and household stress, and limited social support.^10^ Digital interventions offer a promising solution as they can be completed remotely and flexibly, and reduce financial, travel and work-related costs.^11^ Digital interventions can be delivered with human support (guided) or as self-directed programs (unguided). Evidence from the broader literature suggests that both approaches can improve parenting and child outcomes,^12^^,^^13^ although unguided interventions are typically more scalable and cost-effective, whereas guided interventions often achieve higher levels of engagement and adherence^14^ and greater effectiveness in some contexts.^15^ However, little is known about the optimal level of support required in digital parent-focused interventions targeting adolescent health behaviours. Our recent systematic review identified a lack of such programs, and only one designed specifically for parents experiencing socio-economic disadvantage.^8^

The Multiphase Optimisation Strategy (MOST) offers a novel framework to test multiple intervention components simultaneously and build interventions containing only demonstrably effective components.^15^ Unlike traditional randomised controlled trials (RCTs) which typically evaluate interventions as a whole, MOST enables a more precise approach to intervention development by identifying the most effective and efficient components and combinations for a target population. This approach informs which components should be prioritised, and which can be reduced, eliminated, or replaced, to improve efficacy whilst also considering efficiency, cost and/or scalability.^16^ In this context, efficiency is the extent to which an intervention achieves the desired outcome, while minimising resource demands and participant burden.^17^ Efficient interventions prevent participant fatigue and maximise engagement by ensuring that participant effort is only required for components that meaningfully improve the intended outcomes.^17^

MOST consists of three phases: preparation, optimisation and evaluation. In the preparation phase for this study, we developed a conceptual model based on principles from Social Cognitive Theory and Family Systems Theory, and findings from a survey with parents^18^ and a systematic review^8^ (see Appendix). The model included five intervention components: online learning modules, text messages, tailored feedback, stress management training and telehealth coaching. The online modules were included as the ‘core component’, delivered to all parents, while the four additional components were selected to be experimentally tested. The components were designed to: (1) target modifiable parenting factors (mediators); (2) improve parenting practices, parents' own health behaviours and promote parent engagement with the intervention (proximal outcomes); and (3) ultimately improve adolescent health behaviours (distal outcomes). The components were iteratively co-designed with parents and adolescents living in socio-economically disadvantaged communities. This included two parent co-design workshops, two school-based student focus groups, and a parent–adolescent advisory group who met in-person across the project (atop remote paid engagement opportunities, as needed), to provide ongoing advice on the intervention and trial development.

The present study focuses on the ‘optimisation phase’, in which the individual and combined performance of components are tested, and results are used to guide assembly of an ‘optimised intervention package’ that achieves the greatest improvement in the target outcome, with the least resource consumption or burden. The primary aim was to test the individual and synergistic effects of the four components (text messages, tailored feedback, stress management training and telehealth coaching) for improving parental encouragement of adolescent health behaviours among parents living in socio-economically disadvantaged communities over 3-months. The second aim was to assemble an optimised intervention in which all components are effective and impose the least possible burden to parents. In the evaluation phase of MOST, this optimised parent intervention will be trialled in a cluster RCT, as an accompaniment to the existing *Health4Life* school-based intervention,^19^ since parent interventions are known to be most effective when combined with a youth-based component.^8^

## Methods

### Trial design

A 2 × 2 × 2 × 2 (i.e., four factors with two levels each; see Table 1) full factorial trial was implemented among parents living in socio-economically disadvantaged areas of New South Wales (NSW), Australia in 2025. Ethical approval for the study was approved by the University of Sydney (2024/HE001483) and the University of Newcastle (R-2025-0006). Permission was also obtained from the State Education Research Applications Process (SERAP 2024249) to promote the study via NSW government schools. This report follows the Consolidated Standards of Reporting Trials (CONSORT) guidelines, including extensions for factorial trials, and was prospectively registered (ACTRN12624001492549). Further details have been reported in the published protocol.^20^

**Table 1:** Experimental conditions in 2^4^ factorial design.

Condition	Online modules (Core Component)	Tailored feedback	Stress management	Text messages	Health coaching	N randomised
1	**Yes**	**Yes**	**Yes**	**Yes**	**Yes**	18
2	**Yes**	**Yes**	**Yes**	**Yes**	No	19
3	**Yes**	**Yes**	**Yes**	No	**Yes**	19
4	**Yes**	**Yes**	**Yes**	No	No	19
5	**Yes**	**Yes**	No	**Yes**	**Yes**	18
6	**Yes**	**Yes**	No	**Yes**	No	20
7	**Yes**	**Yes**	No	No	**Yes**	19
8	**Yes**	**Yes**	No	No	No	18
9	**Yes**	No	**Yes**	**Yes**	**Yes**	19
10	**Yes**	No	**Yes**	**Yes**	No	19
11	**Yes**	No	**Yes**	No	**Yes**	19
12	**Yes**	No	**Yes**	No	No	19
13	**Yes**	No	No	**Yes**	**Yes**	19
14	**Yes**	No	No	**Yes**	No	19
15	**Yes**	No	No	No	**Yes**	19
16	**Yes**	No	No	No	No	19

Note: N excludes the 1 case that withdraw after randomisation but includes the 10 fraudulent cases that were removed from analysis.

### Participants

Recruitment occurred via multiple channels, including social media, schools, sporting clubs, community organisations, and Local Health Districts (e.g. hospitals and community health centres). Interested parents completed an online screener to assess their eligibility against the inclusion criteria: parent/caregiver of an 11-15-year-old, reflecting the early to middle adolescent years when parental influence remains strong; living in a disadvantaged area of NSW (Australian Bureau of Statistics' Index of Relative Socio-economic Disadvantage; IRSD); access to the internet at home or another suitable location (required to engage with the online intervention); and willing to provide informed consent. The IRSD is an area-level measure that summarises multiple indicators of social and economic disadvantage, including low income, low educational attainment, unemployment, and low-skilled occupations. Participants were required to reside in suburbs classified within the bottom two quintiles, representing the 40% most disadvantaged areas in NSW. To minimise contamination, only one parent per household was eligible. The eligibility screener asked whether another parent/carer from the household was already participating, and individuals were excluded if this was the case. Information collected during enrolment (e.g. contact details and household information) was also reviewed as part of data quality procedures to identify instances of multiple participants originating from the same household. The study was conducted virtually, with no face-to-face interaction.

### Randomisation and masking

A biostatistician with no role in recruitment produced the randomisation scheme using the Blockrand function in R. Randomisation used a blocked random allocation method with randomly selected block sizes to ensure an equal number of participants in each group, while preventing selection bias that can occur when using fixed block sizes. Participants were randomised to 1 of 16 experimental conditions (see Table 1) via the online REDCap system after they completed the baseline assessment, following a data quality check by the research team. As is commonly the case for health intervention trials, blinding to randomised allocation was not possible for participants, research staff and health coaches. However, as all assessments were conducted online, researchers were not required to administer assessments.

### Procedure

Following informed online consent, eligible participants were automatically redirected to complete a 20-min online baseline survey via REDCap. Translated versions of consent forms and surveys were available in Vietnamese and Arabic, the two most common languages other than English in our target communities. Rigorous data quality checks were conducted to identify and exclude fraudulent participants or bots (see Appendix). Only participants who passed the data quality checks were enrolled. Following randomisation, an automated email was sent to participants with details about how to access the core intervention and their allocated components. Parents were sent an automated email invitation to complete a follow-up survey at 3-months post-baseline. The 3-month follow-up was selected to assess proximal changes in parenting practices following completion of the 6-7-week intervention, consistent with the optimisation objectives of the MOST framework and previous parenting intervention research that used our primary outcome.^21^ Email, text messages and phone call reminders were used to enhance retention. Participants were reimbursed a AUD$30 e-gift voucher for each survey. At the end of the intervention period, parents were invited to provide feedback about the program via a brief survey. Participants were advised to contact the lead researcher (KEC) or the University of Sydney's HREC to report adverse events, should they occur.

### The *Health4Life Parents & Teens* intervention (core component)

All enrolled parents received access to the core intervention via the study website (https://parentsandteens.org.au/). This comprised six online learning modules, one module per risk factor: screen time and social media, smoking and vaping, alcohol, sleep, healthy eating, and physical activity (see Appendix). The self-directed modules aimed to empower parents to support their adolescent child to adopt healthy behaviours by enhancing parental self-efficacy and improving knowledge. Modules were approximately 20-min long and were designed to be completed once per week, over a 6-week period. Subsequent modules became available on a weekly basis (one per week) to allow time for parents to consolidate learning and delivery of other intervention components to occur. The modules included a podcast-style video (∼12 min) with a health professional, followed by an interactive summary of recommended parenting strategies and a goal setting activity. Content focused on promoting healthy role-modelling, collaborative and consistent rule-setting, parental monitoring, and open communication (see protocol for further detail).^20^ A strengths-based approach was adopted, with a focus on building upon existing family strengths and fostering positive behaviour change without judgement. The intervention incorporated accessibility features, including mobile-friendly delivery, real-time text translation and ‘text to speech’ support.

### Additional components (factors)

Participants were randomly assigned to receive up to four additional intervention components, each with two levels—‘on’ (received the component) or ‘off’ (did not receive the component; see Table 1).

#### Tailored feedback

Parents randomised to this component were given feedback about their parenting practices in relation to the Big 6 risk behaviours via the study website, before completing their weekly online learning module. Each week, parents were prompted to answer one parenting-related question, (aligning with the online module they were about to begin, e.g. “sleep”), derived from our primary outcome.^22^ Based on their responses (never, rarely, sometimes, often), parents received strengths-based feedback and motivational advice about their encouragement of their child's health habits (see Appendix).

#### Stress management training

Parents randomised to this component were given access to an additional online module about managing stress, which aimed to improve family dynamics and reduce household stress and negative emotion. Key content covered the physical, psychological, and social impacts of chronic stress, work-life balance, identifying and responding to stress triggers, managing financial stress, and self-care. Content was delivered via a 12-minute video podcast featuring a registered psychologist, followed by an interactive summary of strategies and goal setting.

#### Text messages

Parents randomised to this component received text messages, which aimed to provide social support (general encouragement and reinforcement) and encourage engagement with the core intervention. Text messages were semi-personalised (e.g. name) and automated via our study website, containing hyperlinks to prompt interaction with the website (see Appendix). Messages were triggered by interaction events with the core intervention (e.g. starting a module, completing or not completing a module). Participants received 2–3 text messages per week, staggered throughout a 7-day period.

#### Telehealth coaching

Parents randomised to this component were offered weekly coaching calls with a trained non-specialist health coach (research assistants with minimum undergraduate public health training). Coaching aimed to provide supportive accountability and emotional (e.g. encouragement, empathy) and practical (e.g. problem solving, goal setting) social support. Health coaches received approximately 5 h of training with a psychologist, covering motivational interviewing, strengths-based and collaborative approaches, understanding readiness to change, active listening and communication skills, and risk management. Parents could choose telephone or video coaching, select a convenient time, and could schedule sessions via the website; alternatively, the coach contacted them directly. The health coach followed a semi-structured manual which covered module progress, and troubleshooting barriers to intervention engagement, or to implementing parenting strategies and goals from the modules. Telehealth coaching calls were recorded, with a random subsample (10%) rated by an experienced postdoctoral researcher (LD; PhD in Psychology) using a fidelity checklist. A bespoke checklist, adapted from previous health coaching and behavioural intervention fidelity measures,^23^^,^^24^ assessed delivery of key session components (e.g. module content, goals, and barrier problem-solving). Content fidelity was calculated as the percentage of required components delivered (0–100%). Coaches were required to maintain ≥90% fidelity, with retraining planned if fidelity fell below this threshold. Additional ratings assessed adherence to the coaching script, quality of delivery (e.g. rapport, empathic communication, and participant involvement), and parent engagement using Likert-style scales.

### Outcomes

The prespecified primary outcome was change from baseline to 3-months in self-reported parental encouragement of their child's health habits, measured using the Health Habits sub-scale of the Parenting to Reduce Adolescent Depression Anxiety Scale (PRADAS).^22^ The sub-scale includes 12-items assessing health-promoting parenting practices related to healthy eating, sleep, physical activity, and substance use, including encouragement, role modelling, practical support, setting expectations and boundaries, and responses to adolescent substance use. Example items include *“I encourage [my teenager] to eat a healthy, balanced diet, including plenty of fresh vegetables and water”* and *“I have good health habits (i.e., healthy diet, regular exercise, responsible use of alcohol) myself”.* Parents responded on a four-point scale ranging from “never” to “often”. Responses were recoded as guideline concordant (1) or non-concordant (0) according to predefined evidence-based scoring criteria,^22^ and summed to yield a total score of 0–12, with higher scores indicating greater guideline concordance. The PRADAS has been validated with Australian parents.^22^ Reliability was established by calculating agreement coefficients suitable for criterion-referenced measures like the PRADAS and in line with the original psychometric study. This value captures the overall consistency of the Health Habits subscale in classifying parents as concordant or non-concordant with the guidelines, and in the total sample the agreement was estimated as 0.93 indicating very high agreement. Parents with more than one adolescent child were instructed to think about only one child when completing the survey. Parents then provided the selected child's name, which was automatically piped into all PRADAS items. As is acceptable in MOST trials, we chose to use a proximal outcome (parental encouragement of adolescent health habits) as the primary outcome, rather than the distal outcome of adolescent risk behaviours.^16^ Importantly, adolescent outcomes will be assessed in the subsequent ‘evaluation phase’, via an RCT with parent-adolescent dyads.

To assess our secondary objective of assembling the most effective intervention that imposes the least possible burden, parents also responded to a question about *“how much effort did it take to engage with …”* each component they were assigned to (5-point scale from ‘no effort at all’ to ‘huge effort’). This item was adapted from an existing Theoretical Framework of Acceptability questionnaire.^25^ Details of the demographic variables collected are provided in the Appendix. Other variables assessed in this study, including potential moderators and mediators not examined in the present paper, are described in the published protocol.^20^ Acceptability of the intervention, suitability of length and ease of completion were assessed by an online self-report feedback survey. Objective data were also collected via the study website to assess parents' engagement with the intervention (e.g. number of modules accessed, number completed).

### Statistical analysis

#### Sample size calculations

In our original protocol, sample size calculations were powered to detect a small effect size of d = 0.27 on the primary outcome based on prior research,^21^ with 80% power and an alpha of 0.05. This resulted in a minimum of 261 participants required and a recruitment target of 389 participants to account for an anticipated rate of 30% attrition at follow-up.

#### Primary analysis

A pre-specified linear mixed effect (multilevel) model was used to assess the effects of each component on change from baseline to 3-month follow-up on the primary outcome. Data were analysed on an intention-to-treat basis and effect coding was used (‘off’ component levels coded as −1, ‘on’ component levels coded as 1) to enable interpretation of the regression coefficients (main effects) as the mean difference from the grand mean of all components. Random effects were modelled for each participant, whereas time and component levels were modelled as fixed effects. A multiplicative interaction between time and each component was included to investigate significant change in the outcome over time. Likewise, all potential 2-, 3-, and 4-way multiplicative interactions between components with time were included in a full model to identify synergistic or antagonistic effects on the primary outcome. Parent age was included as a covariate a priori. All cases with baseline data were included, and missingness at follow-up was handled via maximum likelihood.

A systematic decision-making process was undertaken^16^ to achieve the secondary objective of assembling an intervention that maximises improvements in the primary outcome, while minimising parent burden. If a time-by-component interaction was deemed important (p < 0.10) in the desired direction, then the corresponding component was provisionally placed in the screened-in set; otherwise, the component was placed in the screened-out set. Linear hypothesis tests were used to compare selected time-by-component interaction coefficients. Next, all interactions were examined, from lower to higher order. Any effects that were considered important were added into a parsimonious prediction model (see Appendix). The parsimonious model was used to estimate the predicted change in parental encouragement of adolescent health habits for selected intervention conditions. Finally, average parent-reported perceived effort associated with completing each component was examined for each combination of components in the screened-in set using the Wilcoxon signed rank test. Estimated improvement in parental encouragement of health habits was calculated based on the parsimonious regression model. The optimised intervention package was selected according to the prespecified optimisation objective of achieving the greatest improvement in the primary outcome with the lowest possible parent burden. Estimated intervention effects and parent-reported effort were considered jointly when identifying the optimal package. All analyses were conducted in R version 4.4.2. No formal data monitoring committee was established. The principal investigator (KEC) and trial co-ordinator (LD) monitored the trial and any adverse events, with regular review by the wider investigator team.

### Changes to trial protocol

Following trial commencement (but prior to any follow-up assessments), the threshold for detecting component effects was revised (from alpha of 0.05 to 0.10). This aligns with MOST guidance, which emphasises an exploratory, rather than confirmatory, approach during early phases of intervention development.^16^ Using a more liberal alpha is common in optimisation trials^26^ as it reduces Type II error, increases sensitivity to detect meaningful effects during component screening, and helps avoid prematurely discarding potentially effective components. Power calculations were updated using alpha = 0.10 while retaining 80% power to detect an effect size of d = 0.27 on the primary outcome. Under these parameters, an estimated 205 parent participants at the follow-up were required to explore time-by-component, 2-way, and 3-way interactions. To ensure balanced allocation across experimental conditions, we required 208 participants (i.e., 13 per condition). To account for approximately 30% attrition, our revised recruitment target at the 3-month follow-up was 298 participants.

### Role of the funding source

The funders had no role in study design, data collection, data analysis, data interpretation, writing of the report, or decision to submit for publication.

## Results

Between April 23 and October 22, 2025, we recruited and randomised 303 participants. One participant (condition 7) withdrew after randomisation, and 10 cases were removed during analysis due to evidence of disingenuous patterns of responding, resulting in a final baseline sample of 292 parents. The follow-up survey was administered 3-months post-baseline from July 27, 2025 to January 28, 2026. Retention at follow-up was 79% (n = 231). Participant flow is shown in Fig. 1. Participants were primarily female at birth (87%; n = 253) with a mean age of 43.02 years (SD = 6.41). The most commonly reported household income bracket was AUD$45,001–$120,000 (42%; n = 122) and over half (55.1%) reported at least one chronic condition (e.g. high blood pressure, Type 2 diabetes). Full participant characteristics for the whole sample, and by component, are in Table 2. No adverse events occurred.

**Fig. 1:**
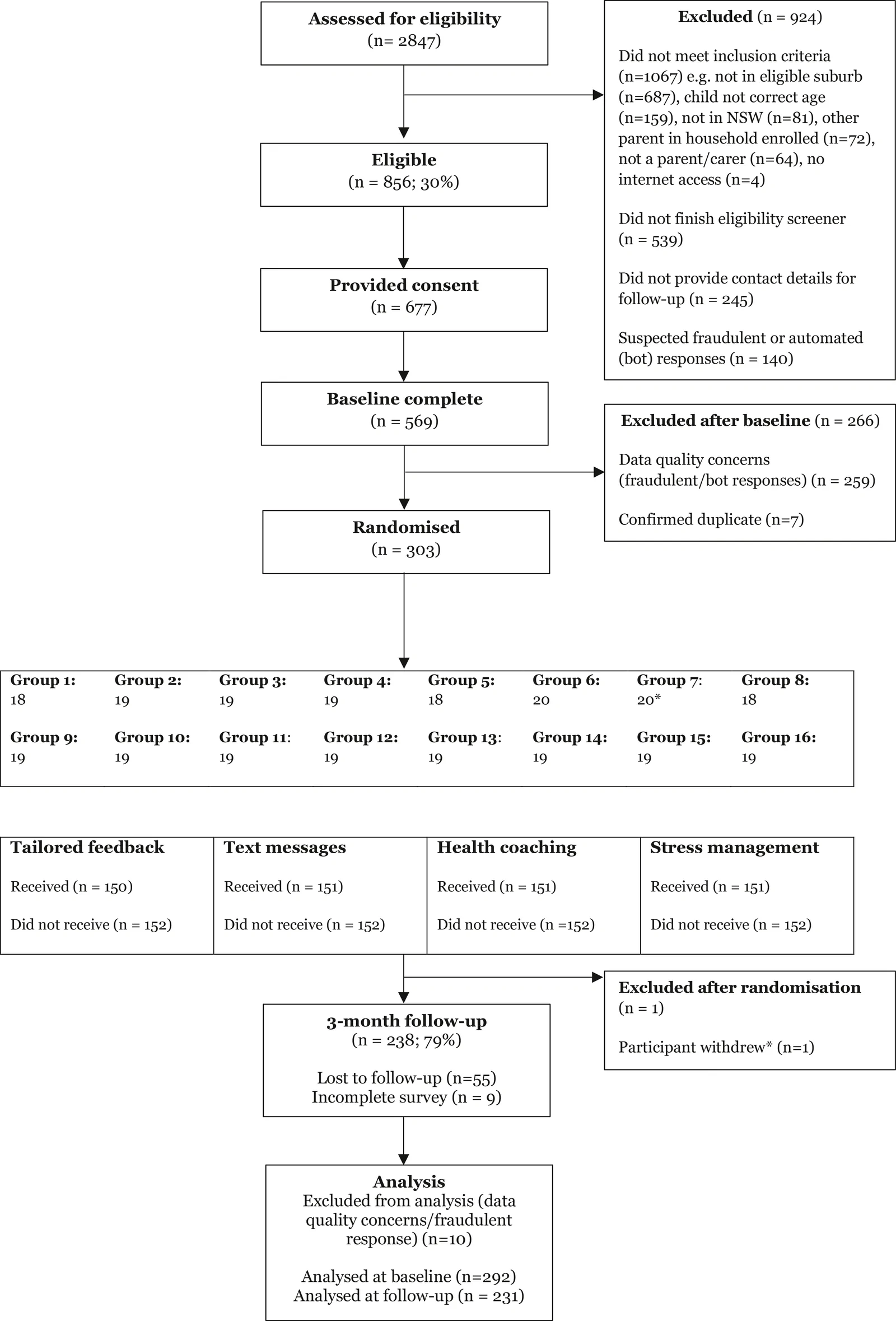
CONSORT flow.

**Table 2:** Baseline characteristics in total sample and by experimental condition.

	Total sample n = 292		Feedback		Text		Coaching		Stress	
			On	Off	On	Off	On	Off	On	Off
**Age** (M, SD)	43.02	(6.41)	43.9 (6.9)	42.2 (5.8)	43.3 (6.3)	42.7 (6.5)	42.9 (5.4)	43.1 (7.3)	43 (6.7)	43.1 (6.2)
**Sex at birth** (n, %)										
Male	38	13%	17 (11.6%)	21 (14.5%)	24 (16.3%)	14 (9.7%)	20 (13.7%)	18 (12.3%)	19 (13.2%)	19 (12.8%)
Female	253	87%	128 (87.8%)	124 (85.5%)	123 (83.7%)	130 (89.7%)	126 (86.3%)	127 (87.0%)	125 (86.8%)	128 (86.5%)
Prefer not to say	1	0.3%	1 (0.7%)	0 (0.0%)	0 (0.0%)	1 (0.7%)	0 (0.0%)	1 (0.7%)	0 (0.0%)	1 (0.7%)
**Gender identity** (n, %)										
Man	39	13%	17 (11.6%)	22 (15.2%)	24 (16.3%)	15 (10.3%)	21 (14.4%)	18 (12.3%)	19 (13.2%)	20 (13.5%)
Woman	249	85%	127 (86.4%)	122 (84.1%)	122 (83%)	127 (87.6%)	124 (84.9%)	125 (85.6%)	123 (85.4%)	126 (85.1%)
Non-binary/gender fluid	3	1%	2 (1.4%)	1 (0.7%)	1 (0.7%)	2 (1.4%)	1 (0.7%)	2 (1.4%)	2 (1.4%)	1 (0.7%)
Prefer not to answer	1	0.3%	1 (0.7%)	0 (0%)	0 (0%)	1 (0.7%)	0 (0%)	1 (0.7%)	0 (0%)	1 (0.7%)
**Country of birth** (n, %)										
Australia	224	77%	110 (74.8%)	114 (78.6%)	114 (77.6%)	110 (75.9%)	112 (76.7%)	112 (76.7%)	114 (79.2%)	110 (74.3%)
Other country	68	23%	37 (25.2%)	31 (21.4%)	33 (22.4%)	35 (24.1%)	34 (23.3%)	34 (23.3%)	30 (20.8%)	38 (25.7%)
**Language other than English spoken at home** (n, %)										
Yes	53	19%	29 (20.6%)	24 (17.1%)	21 (15%)	32 (22.7%)	30 (21.4%)	23 (16.3%)	22 (15.7%)	31 (22%)
No	228	81%	112 (79.4%)	116 (82.9%)	119 (85%)	109 (77.3%)	110 (78.6%)	118 (83.7%)	118 (84.3%)	110 (78%)
**Highest education** (n, %)										
No school qualification	0	0.0%	0 (0.0%)	0 (0.0%)	0 (0.0%)	0 (0.0%)	0 (0.0%)	0 (0.0%)	0 (0.0%)	0 (0.0%)
Year 9 or below	2	0.7%	0 (0.0%)	2 (1.4%)	0 (0.0%)	2 (1.4%)	0 (0.0%)	2 (1.4%)	1 (0.7%)	1 (0.7%)
Year 10	13	4.5%	7 (4.8%)	6 (4.2%)	6 (4.1%)	7 (4.9%)	4 (2.8%)	9 (6.2%)	5 (3.5%)	8 (5.4%)
Year 11	2	0.7%	0.0 (0.0%)	2 (1.4%)	2 (1.4%)	0 (0.0%)	2 (1.4%)	0 (0.0%)	1 (0.7%)	1 (0.7%)
Year 12	19	6.5%	9 (6.1%)	10 (6.9%)	10 (6.8%)	9 (6.2%)	4 (2.8%)	15 (10.3%)	10 (6.9%)	9 (6.1%)
Certificate I/II	6	2.1%	3 (2.0%)	3 (2.1%)	2 (1.4%)	4 (2.8%)	2 (1.4%)	4 (2.7%)	4 (2.8%)	2 (1.4%)
Certificate III/IV	51	17.5%	25 (17.0%)	26 (18.1%)	31 (21.1%)	20 (13.9%)	28 (19.3%)	23 (15.8%)	24 (16.7%)	27 (18.4%)
Advanced diploma	47	16.2%	26 (17.7%)	21 (14.6%)	24 (16.3%)	23 (16%)	28 (19.3%)	19 (13%)	30 (20.8%)	17 (11.6%)
Bachelor's degree	79	27.1%	41 (27.9%)	38 (26.4%)	41 (27.9%)	38 (26.4%)	37 (25.5%)	42 (28.8%)	41 (28.5%)	38 (25.9%)
Grad. dip./Grad cert.	18	6.2%	9 (6.1%)	9 (6.2%)	11 (7.5%)	7 (4.9%)	10 (6.9%)	8 (5.5%)	8 (5.6%)	10 (6.8%)
Postgraduate degree	54	18.6%	27 (18.4%)	27 (18.8%)	20 (13.6%)	34 (23.6%)	30 (20.7%)	24 (16.4%)	20 (13.9%)	34 (23.1%)
Cert. not further defined	0	0.0%	0 (0.0%)	0 (0.0%)	0 (0.0%)	0 (0.0%)	0 (0.0%)	0 (0.0%)	0 (0.0%)	0 (0.0%)
Unsure/Prefer not to say	0	0.0%	0 (0.0%)	0 (0.0%)	0 (0.0%)	0 (0.0%)	0 (0.0%)	0 (0.0%)	0 (0.0%)	0 (0.0%)
**Employment** (n, %)										
Full-time	126	44%	60 (41.7%)	66 (47.1%)	68 (47.6%)	58 (41.1%)	67 (47.9%)	59 (41%)	60 (43.2%)	66 (45.5%)
Part time/casual	103	36%	48 (33.3%)	55 (39.3%)	53 (37.1%)	50 (35.5%)	50 (35.7%)	53 (36.8%)	49 (35.3%)	54 (37.2%)
Unemployed	7	2.5%	5 (3.5%)	2 (1.4%)	3 (2.1%)	4 (2.8%)	4 (2.9%)	3 (2.1%)	5 (3.6%)	2 (1.4%)
Student	7	2.5%	6 (4.2%)	1 (0.7%)	3 (2.1%)	4 (2.8%)	4 (2.9%)	3 (2.1%)	3 (2.2%)	4 (2.8%)
Retired	3	1.1%	2 (1.4%)	1 (0.7%)	0 (0.0%)	3 (2.1%)	0 (0.0%)	3 (2.1%)	2 (1.4%)	1 (0.7%)
Unable to work	8	2.8%	7 (4.9%)	1 (0.7%)	5 (3.5%)	3 (2.1%)	3 (2.1%)	5 (3.5%)	5 (3.6%)	3 (2.1%)
Home duties	27	9.5%	13 (9%)	14 (10%)	10 (7%)	17 (12.1%)	11 (7.9%)	16 (11.1%)	13 (9.4%)	14 (9.7%)
Other	3	1.1%	3 (2.1%)	0 (0.0%)	1 (0.7%)	2 (1.4%)	1 (0.7%)	2 (1.4%)	2 (1.4%)	1 (0.7%)
**Household income** (n, %)										
Nil income–$18,200	9	3.1%	6 (4.1%)	3 (2.1%)	6 (4.1%)	3 (2.1%)	6 (4.2%)	3 (2.1%)	7 (4.9%)	2 (1.4%)
$18,201–$45,000	33	11%	18 (12.2%)	15 (10.6%)	18 (12.2%)	15 (10.6%)	13 (9%)	20 (13.9%)	16 (11.3%)	17 (11.6%)
$45,001–$120,000	122	42%	60 (40.8%)	62 (44%)	60 (40.8%)	62 (44%)	59 (41%)	63 (43.8%)	61 (43%)	61 (41.8%)
$120,001–$180,000	64	22%	33 (22.4%)	31 (22%)	33 (22.4%)	31 (22%)	37 (25.7%)	27 (18.8%)	33 (23.2%)	31 (21.2%)
$180,001 and over	38	13%	20 (13.6%)	18 (12.8%)	20 (13.6%)	18 (12.8%)	19 (13.2%)	19 (13.2%)	17 (12%)	21 (14.4%)
Prefer not to say	22	7.6%	10 (6.8%)	12 (8.5%)	10 (6.8%)	12 (8.5%)	10 (6.9%)	12 (8.3%)	8 (5.6%)	14 (9.6%)
**Marital status** (n, %)										
Single	45	15%	20 (13.7%)	25 (17.2%)	22 (15%)	23 (16%)	19 (13.1%)	26 (17.8%)	23 (16.1%)	22 (14.9%)
Married	151	52%	78 (53.4%)	73 (50.3%)	76 (51.7%)	75 (52.1%)	88 (60.7%)	63 (43.2%)	75 (52.4%)	76 (51.4%)
Divorced	37	13%	17 (11.6%)	20 (13.8%)	23 (15.6%)	14 (9.7%)	12 (8.3%)	25 (17.1%); 4:	14 (9.8%)	23 (15.5%)
De facto	30	10%	13 (8.9%)	17 (11.7%)	15 (10.2%)	15 (10.4%)	13 (9%)	17 (11.6%)	17 (11.9%)	13 (8.8%)
Never married	1	0.3%	1 (0.7%)	0 (0.0%)	0 (0.0%)	1 (0.7%)	1 (0.7%)	0 (0.0%)	0 (0.0%)	1 (0.7%)
Widowed	0	0.0%	0 (0.0%)	0 (0.0%)	0 (0.0%)	0 (0.0%)	0 (0.0%)	0 (0.0%)	0 (0.0%)	0 (0.0%)
Separated	23	7.9%	13 (8.9%)	10 (6.9%)	10 (6.8%)	13 (9%)	11 (7.6%)	12 (8.2%)	11 (7.7%)	12 (8.1%)
Prefer not to say	4	1.4%	4 (2.7%)	0 (0.0%)	1 (0.7%)	3 (2.1%)	1 (0.7%)	3 (2.1%)	3 (2.1%)	1 (0.7%)
**No. of children** (n, %)										
1	57	20%	26 (17.7%)	31 (21.4%)	25 (17%)	32 (22.1%)	21 (14.4%)	36 (24.7%)	26 (18.1%)	31 (20.9%)
2	118	40%	64 (43.5%)	54 (37.2%)	58 (39.5%)	60 (41.4%)	59 (40.4%)	59 (40.4%)	61 (42.4%)	57 (38.5%)
3	68	23%	29 (19.7%)	39 (26.9%)	37 (25.2%)	31 (21.4%)	33 (22.6%)	35 (24%)	28 (19.4%)	40 (27%)
4	28	9.6%	17 (11.6%)	11 (7.6%)	17 (11.6%)	11 (7.6%)	19 (13%)	9 (6.2%)	17 (11.8%)	11 (7.4%)
5	13	4.5%	7 (4.8%)	6 (4.1%)	6 (4.1%)	7 (4.8%)	9 (6.2%)	4 (2.7%)	8 (5.6%)	5 (3.4%)
6+	8	2.7%	4 (2.7%)	4 (2.8%)	4 (2.7%)	4 (2.8%)	5 (3.4%)	3 (2.1%)	4 (2.8%)	4 (2.7%)
**Any chronic condition** (n, %)										
Yes	147	50.3%	81 (55.1%)	66 (45.5%)	74 (50.3%)	73 (50.3%)	76 (52.1%)	71 (48.6%)	74 (51.4%)	73 (49.3%)
No	145	49.6%	66 (44.9%)	79 (54.5%)	73 (49.7%)	72 (49.7%)	70 (47.9%)	75 (51.4%)	70 (48.6%)	75 (50.7%)
**Food insecurity** (n, %)										
Yes	69	24%	35 (24.3%)	34 (23.6%)	32 (22.2%)	37 (25.7%)	40 (28%)	29 (20%)	39 (27.7%)	30 (20.4%)
No	211	73%	105 (72.9%)	106 (73.6%)	108 (75%)	103 (71.5%)	99 (69.2%)	112 (77.2%)	99 (70.2%)	12 (76.2%)
Prefer not to say	8	2.8%	4 (2.8%)	4 (2.8%)	4 (2.8%)	4 (2.8%)	4 (2.8%)	4 (2.8%)	3 (2.1%)	5 (3.4%)
**Parental stress (M, SD)**	43.8 (10.0)		44.1 (10.8)	43.5 (9.2)	44.9 (9.8)	42.7 (10.2)	42.6 (9.5)	45.0 (10.4)	44.6 (10.2)	43.1 (9.8)

Tailored feedback (*B* = 0.21, p = 0.054, mean difference = 0.42, 95% CI = −0.01 to 0.85, Cohen's d = 0.26) and telehealth coaching (*B* = 0.31, p = 0.0051, mean difference = 0.62, 95% CI = 0.19 to 1.0, Cohen's d = 0.38) significantly improved parental encouragement of health habits (e.g. PRADAS scores) from baseline to 3-months (see Table 3). Therefore, both components were tentatively placed in the screened-in set (i.e., retained for further consideration during optimisation). Subsequent linear hypothesis tests comparing the two component-by-time interaction coefficients indicate that there was no evidence of a significant difference in change over time when receiving tailored feedback or telehealth coaching (X^2^ = 0.44, df = 1, p = 0.51). There was also a significant time-by-component interaction for text messaging (*B* = −0.33, p = 0.0026, mean difference = −0.66, 95% CI = −1.09 to −0.23, Cohen's d = −0.41), however it reduced parental encouragement from baseline to 3-months. Therefore, we tentatively screened-out text messages. Stress management was also screened-out as there was no evidence of a significant time-by-component interaction. Next, we reconsidered these decisions in light of important interaction effects among the components. We particularly examined interactions that included feedback and coaching to determine whether any of the other components should be included, because they boosted or reduced the effect of tailored feedback or telehealth coaching. There was no evidence of any significant 2-, 3-, or 4-way interactions between time and different combinations of the chosen components.

**Table 3:** Results of the full regression model testing the effects of all intervention components on the primary outcome.

	B	SE	T value	p value
Baseline				
Intercept	5.41	0.61	8.93	<0.0001
Time	0.48	0.11	4.38	<0.0001
Parent age	0.03	0.01	2.35	0.020
Feedback	0.09	0.10	0.88	0.38
Text	0.15	0.10	1.56	0.12
Coaching	−0.10	0.10	−1.04	0.30
Stress	−0.06	0.10	−0.57	0.57
Feedback × Stress	0.03	0.10	0.28	0.78
Feedback × Text	−0.07	0.10	−0.70	0.48
Feedback × Coaching	−0.21	0.10	−2.18	0.03
Text × Stress	0.01	0.10	0.13	0.89
Coaching × Stress	0.05	0.10	0.54	0.59
Text × Coaching	0.03	0.10	0.29	0.77
Feedback × Text × Stress	0.06	0.10	0.66	0.51
Feedback × Coaching × Stress	0.03	0.10	0.28	0.78
Feedback × Text × Coaching	−0.10	0.10	−1.00	0.32
Text × Coaching × Stress	−0.05	0.10	−0.61	0.54
Feedback × Text × Coaching × Stress	−0.16	0.10	−1.66	0.10
3-months				
Time × Feedback	0.21	0.11	1.94	**0.054**
Time × Stress	0.03	0.11	0.31	0.75
Time × Text	−0.33	0.11	−3.05	**0.0026**
Time × Coaching	0.31	0.11	2.83	**0.0051**
Time × Feedback × Stress	0.06	0.11	0.51	0.61
Time × Feedback × Text	0.16	0.11	1.48	0.14
Time × Feedback × Coaching	0.11	0.11	0.97	0.33
Time × Text × Stress	0.02	0.11	0.21	0.83
Time × Coaching × Stress	−0.13	0.11	−1.22	0.22
Time × Text × Coaching	−0.13	0.11	−1.16	0.25
Time × Feedback × Text × Stress	0.03	0.11	0.26	0.80
Time × Feedback × Coaching × Stress	0.02	0.11	0.19	0.85
Time × Feedback × Text × Coaching	0.11	0.11	1.05	0.30
Time × Text × Coaching × Stress	0.12	0.11	1.11	0.27
Time × Feedback × Text × Coaching × Stress	0.14	0.11	1.27	0.21

Note: due to the use of coding associated with the components (e.g. −1 and 1), the beta coefficients cannot be interpreted strictly as the total mean difference between components, but instead represent half of the observed effect (the full effect can be calculated by doubling the beta coefficient).

Bold p-values indicate components meeting the prespecified threshold of p < 0.10.

Table 4 shows the expected change from baseline to 3-months in the primary outcome for each combination of components in the screened-in set based on a parsimonious regression model (i.e., screened out components were removed from the model; see Appendix). Condition 15 (telehealth coaching only) produced the greatest expected improvement in the primary outcome but telehealth coaching was also associated with the greatest parent-perceived effort among those who received both components (mean out of 5 = 2.4, SD = 1.2). Condition 8 (tailored feedback only) had comparable improvement on the primary outcome, but tailored feedback was also perceived as significantly less effort among parents who received both components (mean out of 5 = 2.1, SD = 1.0; Wilcoxon signed rank V = 48 p = 0.03). Therefore, the intervention package containing tailored feedback plus the online modules (core component) best achieved our optimisation criterion.

**Table 4:** Predicted change in parental encouragement of adolescent health habits from baseline to 3-months for selected intervention conditions following the screening process.

Condition	Online Modules	Tailored Feedback	Stress Management	Text Messages	Telehealth coaching	PRADAS Score (95% CI)	Predicted change from baseline (95% CI)
**1**	**Yes**	No	No	No	No	6.5 (5.7 to 7.3)	−0.09 (−0.9 to 0.7)
8	**Yes**	**Yes**	No	No	No	7.3 (6.5 to 8.1)	0.7 (−0.1 to 1.5)
15	**Yes**	No	No	No	**Yes**	7.8 (7.1 to 8.6)	1.2 (0.5 to 2.0)
7	**Yes**	Yes	No	No	**Yes**	7.9 (7.1 to 8.6)	1.3 (0.5 to 2.0)

Conditions 8 and 15 correspond to the intervention conditions representing the components retained following the screening process (tailored feedback and health coaching, respectively). Conditions 1 (online modules only) and 7 (tailored feedback plus health coaching) are included for comparison.

Process data (collected via the intervention website) indicated that parents completed a mean of 2.02 out of 6 online modules (SD = 2.65). A total of 105 telehealth coaching sessions were delivered, reaching 52.3% of randomised participants at least once. Call duration ranged from 3.6 to 57.4 min (mean = 11.3 min; SD = 5.23). Average fidelity to the telehealth coaching protocol was 91.9% throughout the study and no retraining was required. A total of 85 parents provided feedback about the program. Most respondents had a “good” or “very good” experience using the program (n = 83; 97.7%), thought the program was “about right” in length (n = 77; 90.6%) and found it “somewhat easy” or “very easy” to find time to do the program (n = 68; 80%).

## Discussion

This study used a novel optimisation framework to develop a digital health intervention for parents living in socio-economically disadvantaged communities in Australia, to improve their encouragement of adolescent health behaviours. We used a factorial design to systematically test four intervention components, and combinations of components, making it possible to eliminate more burdensome, and less impactful components. Results suggested that the combination of the online learning modules (core component) and tailored feedback about parenting strategies best met our optimisation criterion of a maximally effective and minimally burdensome intervention for parents.

Telehealth coaching aimed to provide supportive accountability and social support to parents, which is increasingly recognised as an important BCT for health interventions targeting multiple behaviours.^8^^,^^26^ Research shows that clinician or coaching support improves the effectiveness of, and adherence to, digital interventions.^15^^,^^24^^,^^27^^,^^28^ As such, this study aimed to experimentally test whether regular human support provides additional benefits for parents, and at what expense to their time and capacity. Our results showed that weekly coaching calls were effective in improving parental encouragement of adolescent health habits. However, coaching was screened out due to higher perceived effort from parents. Additionally, telehealth coaching is resource-intensive and costly to deliver, requiring substantial staff time even when delivered remotely by non-specialist health coaches, which significantly limits its scalability.^28^ Future research should explore how coaching can be delivered with less burden, potentially by leveraging emerging technologies such as artificial intelligence (AI).^28^ For these reasons, exploring alternative ways to effectively deliver human-like support (e.g. conversational agents) has been highlighted as a priority issue for digital health intervention research,^28^ however careful consideration of the risks of AI and implications of not using trained researchers or clinicians is needed.

Our study demonstrated that tailored feedback was effective in improving parental encouragement over time. Personalised feedback is commonly used in health interventions to increase engagement, provide motivation and prompt greater self-assessment of behaviour.^8^ Indeed, a 2023 umbrella review of digital health interventions^28^ found strong evidence to support the inclusion of self-regulatory BCTs (goal setting, self-monitoring, and feedback) and use of personalised approaches. Our results indicated that telehealth coaching performed marginally better than tailored feedback, but not significantly so. Importantly, there were no significant higher-order interaction effects. That is, including tailored feedback *and* telehealth coaching did not have a significant synergistic effect over and above providing each component alone, which challenges the assumption that ‘more’ is better. When parents' perceived effort was considered, tailored feedback was rated as less burdensome than telehealth coaching. Our findings are broadly consistent with existing research. For example, our systematic review of parent-based interventions targeting adolescent health behaviours (delivered both face-to-face and digitally) found that personalised feedback and social support were associated with beneficial intervention effects. However, unlike most previous studies in this field, which have largely evaluated intervention packages, the MOST framework enabled the effects of individual components to be evaluated independently. This provided evidence not only about what works, but also which components should be retained in an optimised intervention based on effectiveness and participant burden. The resulting optimised intervention, comprising brief, automated tailored feedback and the core online modules, represents a low-effort, feasible and highly scalable digital intervention to support parents to improve adolescent health risk behaviours.

Interestingly, text messages had an unexpected negative effect, reducing parental encouragement over time. Text messages were sent to parents 2–3 times per week to provide encouragement and remind parents to engage with the online modules. Although prior research has shown the benefits of text messages in parent interventions,^29^^,^^30^ in our sample of Australian parents living in disadvantaged areas, we found clear evidence that this component not only failed to add value but was counterproductive. Despite the text messages being developed with input from parents, it is possible that this co-design process was not intensive enough, that the messages created additional pressure for already time-poor parents, or that parents were already receiving a high volume of messages and became disengaged or distrusting. It is also possible that the text messages were too generic and did not provide sufficient social support. Examining qualitative data collected from parents, and objective engagement data, will provide further information about how text messages were received and why they had an unintended negative effect. Nonetheless, these findings reinforce the value of MOST and the importance of selecting, or in this case, not selecting, components based on empirical performance.

Our findings demonstrated that stress management training had no effect on our primary outcome. Stress management was included as a component due to the higher rates of household stress commonly observed in families experiencing disadvantage,^31^ and evidence that reducing stress improves parenting practices and youth outcomes.^32^ In our conceptual model, parental stress was hypothesised to be one mechanism through which the intervention could influence parental encouragement of adolescent health behaviours. However, the absence of an effect may reflect the fact that, for families experiencing multiple ongoing stressors, increasing awareness of stress and coping strategies does not necessarily translate into measurable improvements in parenting practices within the timeframe of the intervention. The stress module was offered as the last of seven weekly modules. It is therefore also possible that this component was impacted by participant drop-out and/or parent fatigue. Future research will be useful for determining engagement with, and acceptability of, the stress management component, and could inform ongoing optimisation for future iterations of *Health4Life Parents & Teens* (e.g. whether delivering this content earlier, more frequently, or in a different format, is more effective). Additionally, examining mechanisms of change across all components, based on our conceptual model, has potential to advance knowledge for the field. Factorial mediation analysis, well-suited to MOST trials, can examine how individual intervention components work, even in the absence of significant effects,^33^ while moderation analyses may identify subgroups (by parent characteristics, e.g. income, health literacy or family structure) for whom components are more or less effective. Process data could explain and identify predictors of intervention engagement and drop-out, while parent evaluation data has the potential to guide future refinements to the program (e.g. expanded mental health content and greater cultural and family diversity). Together, these insights could support ongoing intervention optimisation and the development of more tailored, potentially adaptive interventions to better meet the needs of diverse parent populations. More broadly, evidence from parent-based interventions targeting adolescent health behaviours remains concentrated in high-income countries, with most studies identified in our systematic review conducted in the United States.^8^ Future research should prioritise optimising and evaluating parent-focused interventions in low- and middle-income settings.

A key strength of this study is the use of a novel methodological framework. This is the first time MOST has been applied to optimise a digital health intervention aimed at supporting parents to improve multiple adolescent health behaviours. Strong participant retention and the co-designed nature of the intervention components further strengthen this study. However, there are some limitations. First, our sample primarily comprised mothers (87%) and was recruited from a single Australian state, which may limit the generalisability of our findings. However, there was some diversity in the sample in terms of country of birth and language spoken at home, and the gender imbalance reflects broader patterns in Australia, where caregiving responsibilities continue to fall disproportionately on women.^34^ Secondly, although all parents resided in socio-economically disadvantaged communities, many held post-secondary qualifications and some reported moderate-to-high household income. This reflects the study's use of an area-level eligibility criterion, which was selected to maximise reach and feasibility while avoiding the potential stigma and barriers associated with individual-level socioeconomic screening. Consequently, the sample may not be representative of families experiencing the most severe forms of individual-level socioeconomic disadvantage. Importantly, the *Health4Life Parents & Teens* intervention was designed to target key parenting strategies that are relevant across a range of socio-economic circumstances. In addition, participation required internet access to engage with the digital intervention. However, internet access is widespread in Australia, and only four individuals (0.4% of those screened) were excluded on this basis. Nevertheless, the findings may be less generalisable to populations experiencing greater levels of digital exclusion.

Another limitation is that our primary outcome was focused on parent self-report, and responses on the primary outcome may have been influenced by social desirability bias, particularly for participants receiving tailored feedback. However, this reflects the deliberate and theoretically grounded nature of tailored feedback interventions, which intentionally rely on self-assessment to guide behaviour change. Additionally, we used a more liberal criterion for statistical significance (p < .10) than is typically used. However, this approach is consistent with the exploratory objectives of the MOST optimisation phase^16^ and was intended to reduce the risk of Type II error and prematurely discarding promising intervention components. Finally, although this study cannot determine whether the optimised parent intervention improves adolescent behaviour, this will be examined in an RCT (ACTRN12626000548336) which is underway as part of the final ‘evaluation phase’ of MOST. The cluster RCT and economic evaluation is being conducted in a new sample of parents and adolescents to evaluate whether combining the optimised *Health4Life Parents & Teens* program with the digital *Health4Life* school-based program is more effective and more cost-effective than usual health education, in modifying the Big 6 adolescent health risk behaviours.

In conclusion, this study used an engineering-inspired, highly efficient framework to yield an optimised, resource-efficient intervention to support parents living in disadvantaged communities to improve adolescent health risk behaviours. It demonstrates the benefits of intervention optimisation approaches, such as those guided by the MOST framework. This study has important implications for other researchers developing behavioural interventions, particularly in settings and populations where resources are constrained.

## Contributors

KEC and LD led the development of this Article and are equal first authors. KEC conceptualised the study. KEC, DL, NCN, LB, MT, CM, LAG, BS, TS, CC, TB, LT, SRP, MS, BP, FK, GJ, ES, SW and LE secured funding for this study. LD, KEC, EH, AH, TF and BO led intervention development. DL, CC, TB, FK and ES recorded video podcasts for the intervention. LD and KEC were responsible for ethics and governance and overall trial coordination, with assistance from EH, and oversight from NCN and DL. KEC and LD supervised staff and students. EH, LD, AH and TF were responsible for recruitment of participants, data collection and quality assurance checks. AH and EH delivered the telehealth coaching component. MS led data analysis and MS, TS and LD were responsible for data monitoring. Data were directly accessed and verified by MS, EH, KC and LD. All authors had full access to all the data. All authors contributed to developing protocols for the study and reviewed, edited, and approved the final version of the manuscript. KC and LD had final responsibility for the decision to submit for publication.

## Data sharing statement

The statistical analysis code and data collected for the study, including de-identified participant data, will be made available to researchers on request to the corresponding author and with appropriate reason, when accompanied by study protocol and analysis plan. Data will be shared after the approval of a proposal by a committee of the current research team, with a signed data access agreement.

## Declaration of interests

KEC, NN, and MT were developers of the school-based ‘Health4Life’ program that accompanies the *Health4Life Parents & Teens* program. The school-based Health4Life program (Program IP) is owned by Climate Schools Pty Ltd, a private company established in 2015 to maximise social well-being. MT and NN are directors and shareholders of Climate Schools Pty Ltd. The Program IP is licensed from Climate Schools Pty Ltd to OurFutures Institute Limited, a not-for-profit entity that distributes the Program IP. MT and NN are cofounders and non-executive board members of the Our Futures Institute. The program developers may receive royalties from commercialisation of Program IP, contingent on the Institute meeting ambitious licensing targets designed to prioritise public benefits.

## Appendix A Supplementary data

Supplementary Materials

CHAMPION_Optimisation Protocol

## References

[1] Baird S., Choonara S., Azzopardi P.S. (2025). A call to action: the second Lancet Commission on adolescent health and wellbeing. Lancet.

[2] Uddin R., Lee E.-Y., Khan S.R., Tremblay M.S., Khan A. (2020). Clustering of lifestyle risk factors for non-communicable diseases in 304,779 adolescents from 89 countries: a global perspective. Prev Med.

[3] Kaikkonen J.E., Mikkilä V., Magnussen C.G., Juonala M., Viikari J.S., Raitakari O.T. (2013). Does childhood nutrition influence adult cardiovascular disease risk?--insights from the Young Finns Study. Ann Med.

[4] AIHW (2020).

[5] Australian Institute of Health and Welfare (2020).

[6] Yap M.B.H., Cheong T.W.K., Zaravinos-Tsakos F., Lubman D.I., Jorm A.F. (2017). Modifiable parenting factors associated with adolescent alcohol misuse: a systematic review and meta-analysis of longitudinal studies. Addiction.

[7] Brown H.E., Atkin A.J., Panter J., Wong G., Chinapaw M.J., van Sluijs E.M. (2016). Family-based interventions to increase physical activity in children: a systematic review, meta-analysis and realist synthesis. Obes Rev.

[8] Champion K.E., Gardner L.A., McCann K. (2022). Parent-based interventions to improve multiple lifestyle risk behaviors among adolescents: a systematic review and meta-analysis. Prev Med.

[9] Ingoldsby E.M. (2010). Review of interventions to improve family engagement and retention in parent and child mental health programs. J Child Fam Stud.

[10] Chacko A., Jensen S.A., Lowry L.S. (2016). Engagement in behavioral parent training: review of the literature and implications for practice. Clin Child Fam Psychol Rev.

[11] Broomfield G., Wade C., Yap M.B.H. (2021). Engaging parents of lower-socioeconomic positions in internet- and mobile-based interventions for youth mental health: a qualitative investigation. Int J Environ Res Public Health.

[12] Dunn A., Alvarez J., Arbon A. (2024). Effectiveness of an unguided modular online intervention for highly anxious parents in preventing anxiety in their children: a parallel group randomised controlled trial. Lancet Reg Health Eur.

[13] Whitaker E., Chessell C., Klapow M., Creswell C. (2025). A systematic review of guided, parent-led digital interventions for preadolescent children with emotional and behavioural problems. Clin Child Fam Psychol Rev.

[14] Day J.J., Sanders M.R. (2018). Do parents benefit from help when completing a self-guided parenting program online? A randomized controlled trial comparing triple P online with and without telephone support. Behav Ther.

[15] Tong L., Panagiotopoulou O.M., Radio M. (2026). Impact of support levels on effectiveness and drop-out of internet-based interventions for depression: network meta-analysis. Br J Psychiatry.

[16] Collins L.M. (2018).

[17] Collins L.M., Nahum-Shani I., Guastaferro K., Strayhorn J.C., Vanness D.J., Murphy S.A. (2024). Intervention optimization: a paradigm shift and its potential implications for clinical psychology. Annu Rev Clin Psychol.

[18] Champion K.E., Hunter E., Gardner L.A. (2023). Parental information needs and intervention preferences for preventing multiple lifestyle risk behaviors among adolescents: cross-sectional survey among parents. JMIR Pediatr Parent.

[19] Champion K.E., Newton N.C., Gardner L.A. (2023). Health4Life eHealth intervention to modify multiple lifestyle risk behaviours among adolescent students in Australia: a cluster-randomised controlled trial. Lancet Digit Health.

[20] Champion K.E., Davidson L., Hunter E. (2025). Optimising a digital intervention to support parents experiencing socio-economic disadvantage to improve adolescent health behaviours: protocol for the Health4Life Parents & Teens factorial trial. Contemp Clin Trials.

[21] Yap M.B.H., Mahtani S., Rapee R.M. (2018). A tailored web-based intervention to improve parenting risk and protective factors for adolescent depression and anxiety problems: postintervention findings from a randomized controlled trial. J Med Internet Res.

[22] Cardamone-Breen M.C., Jorm A.F., Lawrence K.A., Mackinnon A.J., Yap M.B.H. (2017). The parenting to reduce adolescent depression and anxiety scale: assessing parental concordance with parenting guidelines for the prevention of adolescent depression and anxiety disorders. PeerJ.

[23] Newton N.C., Debenham J., Slade T. (2022). Effect of selective personality-targeted alcohol use prevention on 7-year alcohol-related outcomes among high-risk adolescents: a secondary analysis of a cluster randomized clinical trial. JAMA Netw Open.

[24] Spring B., Pfammatter A.F., Scanlan L. (2024). An adaptive behavioral intervention for weight loss management: a randomized clinical trial. JAMA.

[25] Sekhon M., Cartwright M., Francis J.J. (2022). Development of a theory-informed questionnaire to assess the acceptability of healthcare interventions. BMC Health Serv Res.

[26] Spring B., Pfammatter A.F., Marchese S.H. (2020). A factorial experiment to optimize remotely delivered behavioral treatment for obesity: results of the Opt-IN study. Obesity.

[27] Mohr D.C., Cuijpers P., Lehman K. (2011). Supportive accountability: a model for providing human support to enhance adherence to eHealth interventions. J Med Internet Res.

[28] Mair J.L., Salamanca-Sanabria A., Augsburger M. (2023). Effective behavior change techniques in digital health interventions for the prevention or management of noncommunicable diseases: an umbrella review. Ann Behav Med.

[29] Barnes C., Janssen L., Mantach S. (2025). Are text-message based programmes targeting adolescents and their parents an acceptable approach to preventing adolescent e-Cigarette use?. Health Promot J Austr.

[30] Chu J.T.W., Wadham A., Jiang Y. (2019). Effect of MyTeen SMS-based mobile intervention for parents of adolescents: a randomized clinical trial. JAMA Netw Open.

[31] Masarik A.S., Conger R.D. (2017). Stress and child development: a review of the family stress model. Curr Opin Psychol.

[32] Burgdorf V., Szabó M., Abbott M.J. (2019). The effect of mindfulness interventions for parents on parenting stress and youth psychological outcomes: a systematic review and meta-analysis. Front Psychol.

[33] Strayhorn J.C., Collins L.M., Brick T.R. (2022). Using factorial mediation analysis to better understand the effects of interventions. Transl Behav Med.

[34] Hill E, Cooper R, Lee T, Seetahul S (2024). Building a More Equitable Future of Work for Women in NSW: Opportunities for Government Action.

