# Supplementary material for: Optimising a digital parent-based intervention to improve adolescent health risk behaviours in families living in socio-economically disadvantaged communities: findings from the Health4Life Parents & Teens factorial randomised trial
Source: Lancet Reg Health West Pac. 2026 Sep 23;74:101987. doi: 10.1016/j.lanwpc.2026.101987 (PMC13649520; doi:10.1016/j.lanwpc.2026.101987)
Supplement: Supplementary file 1 [file mmc2.pdf]

Contemporary Clinical Trials 154 (2025) 107958

Contents lists available at ScienceDirect

Contemporary Clinical Trials

journal homepage: www.elsevier.com/locate/conclintrial

Optimising a digital intervention to support parents experiencing socio-economic disadvantage to improve adolescent health behaviours: Protocol for the *Health4Life Parents & Teens* factorial trial

Katrina E. Champion [a,b,*,1], Lily Davidson [a,1], Emily Hunter [a], Louise Thornton [a,c], Bonnie Spring [d,e], Bridie Osman [a], Matthew Sunderland [a], Cath Chapman [a], Tracy Burrows [f,g], Tim Slade [a], Stephanie R. Partridge [h,i], Lauren Gardner [a], Belinda Parmenter [j,k], Louise A. Baur [l], Maree Teesson [a], Cathrine Mihalopoulos [m], Alex Haidinger [a], Tara Finn [f,n], Lyra Egan [a], George Johnson [o], Emily Stockings [a], Susan Woolfenden [o,p], Nicola C. Newton [a,2], David R. Lubans [f,n,q,2]

[a] *The Matilda Centre for Research in Mental Health and Substance Use, The University of Sydney, Sydney, NSW, Australia*
[b] *Sydney School of Public Health, The University of Sydney, Sydney, NSW, Australia*
[c] *School of Medicine and Public Health, The University of Newcastle, Callaghan, NSW, Australia*
[d] *Department of Preventive Medicine, Northwestern University Feinberg School of Medicine, Chicago, IL, USA*
[e] *Department of Behavioral Science and Social Medicine, Florida State University College of Medicine, Tallahassee, FL, USA*
[f] *Hunter Medical Research Institute, New Lambton Heights, New South Wales, Australia*
[g] *School of Health Sciences, College of Health, Medicine and Wellbeing, University of Newcastle, Callaghan, NSW, Australia*
[h] *Susan Wakil School of Nursing and Midwifery, The University of Sydney, Sydney, NSW, Australia*
[i] *Charles Perkins Centre, The University of Sydney, Sydney, NSW, Australia*
[j] *School of Health Sciences, University of New South Wales, Sydney, NSW, Australia*
[k] *School of Health, University of the Sunshine Coast, QLD, Australia*
[l] *Children's Hospital Westmead Clinical School, The University of Sydney, Westmead, New South Wales, Australia*
[m] *School of Public Health and Preventative Medicine, Monash University, Australia*
[n] *Centre for Active Living and Learning, College of Human and Social Futures, University of Newcastle, Callaghan, NSW, Australia*
[o] *Sydney Institute for Women, Children and their Families, Sydney Local Health District, Sydney, NSW, Australia*
[p] *Community Paediatric Research Group, Sydney Medical School, University of Sydney, Sydney, NSW, Australia*
[q] *Faculty of Sport and Health Sciences, University of Jyväskylä, Jyväskylä, Finland*

ARTICLE INFO

Keywords:
Parents
Adolescents
Risk behaviours
Digital
Socio-economic disadvantage
Multiphase optimisation strategy

ABSTRACT

Background: Youth experiencing socio-economic disadvantage often have elevated rates of health risk behaviours. Although adolescence is a time of increasing autonomy for youth, parents continue to play a significant role in their health. Parent-based interventions can reduce barriers faced by families experiencing disadvantage and improve adolescent outcomes. However, few such interventions addressing multiple risk behaviours exist for this population. This study aims to: i) systematically test the individual and synergistic effects of four parent-based intervention components, and ii) assemble an optimised intervention in which all components are

* Corresponding author at: The Matilda Centre for Research in Mental Health and Substance Use, The University of Sydney, Sydney, NSW, Australia.
E-mail addresses: katrina.champion@sydney.edu.au (K.E. Champion), lily.davidson@sydney.edu.au (L. Davidson), e.hunter@sydney.edu.au (E. Hunter), louise.thornton@sydney.edu.au (L. Thornton), bonnie.spring@fsu.edu (B. Spring), bridie.osman@sydney.edu.au (B. Osman), matthew.sunderland@sydney.edu.au (M. Sunderland), cath.chapman@sydney.edu.au (C. Chapman), tracy.burrows@newcastle.edu.au (T. Burrows), tim.slade@sydney.edu.au (T. Slade), stephanie.partridge@sydney.edu.au (S.R. Partridge), lauren.gardner@sydney.edu.au (L. Gardner), bparmenter@usc.edu.au (B. Parmenter), louise.baur@sydney.edu.au (L.A. Baur), maree.teesson@sydney.edu.au (M. Teesson), cathy.mihalopoulos@monash.edu (C. Mihalopoulos), alexander.haidinger@sydney.edu.au (A. Haidinger), tara.finn@newcastle.edu.au (T. Finn), lyra.egan@sydney.edu.au (L. Egan), george.johnson@health.nsw.gov.au (G. Johnson), emily.stockings@sydney.edu.au (E. Stockings), susan.woolfenden@health.nsw.gov.au (S. Woolfenden), nicola.newton@sydney.edu.au (N.C. Newton), david.lubans@newcastle.edu.au (D.R. Lubans).
[1] Equally credited first authors.
[2] Equally credited senior authors.

https://doi.org/10.1016/j.cct.2025.107958
Received 13 February 2025; Received in revised form 13 May 2025; Accepted 16 May 2025
Available online 18 May 2025
1551-7144/© 2025 The Authors. Published by Elsevier Inc. This is an open access article under the CC BY license (http://creativecommons.org/licenses/by/4.0/).

effective in improving parental encouragement of adolescent health habits, while imposing the least possible burden on parents.

Methods: Guided by the Multiphase Optimisation Strategy, we will conduct a 2^4 factorial trial with 389 parents of adolescents in Australia. All parents will receive six online modules (screen time, smoking/vaping, alcohol, sleep, food/nutrition, physical activity). Parents will be randomised to receive 1 of 16 combinations of additional components: text messages, tailored feedback, stress management, and health coaching. Parents will complete surveys at baseline and 3-months post-baseline. The primary outcome is change in parental encouragement of adolescent health habits at 3-months (proximal outcome). Parent's health behaviours, self-efficacy, stress, communication and the acceptability and burden (perceived effort to complete) of each component will also be assessed.

Discussion: This study will result in a digital intervention for families experiencing socio-economic disadvantage that is as effective as possible in improving parental encouragement of adolescent health habits, and that can be delivered with minimal burden, which is critical for translation.

Trial registration: The trial was prospectively registered with the Australian New Zealand Clinical Trials Registry (ACTRN12624001492549 date registered: 20/12/2024).

Adolescence is a unique developmental period, characterised by rapid physical, cognitive and social changes, and a critical time for laying the foundations of long-term health and wellbeing [1,2]. Health risk behaviours, such as physical inactivity, poor diet, alcohol use, tobacco smoking, sedentary behaviours (sitting and screen time) and poor sleep (the "Big 6"), often emerge during this life stage and commonly co-occur [3,4]. In Australia, approximately 80 % of adolescents are not sufficiently physically active and 90 % exceed screen time guidelines [5]. However, the prevalence of risk factors is not uniform across populations - youth who experience socio-economic disadvantage are 12 % less likely to be physically active [6] and 22 times more likely to drink at risky levels [7] than their more advantaged peers. Patterns of behaviour established during adolescence often track into adulthood, shaping long-term health trajectories and contributing to the burden of chronic disease across the life course [8]. Addressing these health risk behaviours early in life is therefore essential for not only improving immediate adolescent health, but also for interrupting the broader cycle of disadvantage and preventable disease. Innovative and scalable prevention approaches to address health risk behaviours are urgently needed, particularly for youth experiencing disadvantage [9].

Although numerous interventions to improve youth health risk behaviours exist, they are limited in their impact [10], and do not always align well with best practice recommendations [11]. Additionally, there is a need for greater attention to preventive care models that address multiple risk factors simultaneously [12]. Multiple health behaviour change (MHBC) interventions capitalise on evidence that changing one health behaviour can increase self-efficacy to improve others [13]. In 2018, we developed *Health4Life*, a MHBC intervention targeting the "Big 6" risk factors. It comprises six web-based modules completed by Grade 7 students in health education classes, and a smartphone app. A randomised controlled trial (RCT) found that *Health4Life* improved knowledge about risk factors for up to 2 years [14], modified behavioural intentions [15], and reduced depression and distress in the short-term [16]. While most students found the intervention to be acceptable, students experiencing socio-economic disadvantage found the program less relatable [17]. These findings suggest that more research is needed to translate knowledge into behaviour change, sustain improvements in wellbeing, and better meet the needs of youth who experience disadvantage.

Although adolescence is a time of increasing autonomy, parents remain an important influence on adolescent health [18,19]. For teenagers from disadvantaged backgrounds, research shows that parent engagement continues to be important for positive outcomes even into late adolescence [20]. Research indicates that certain parenting factors and styles are associated with improved adolescent health behaviours. For example, authoritative parental rule-setting (expectations and guidelines set by parents with input from their child, with clear reasoning) [21] can improve youth sleep [22] and reduce screen time [23]; and role-modelling can reduce smoking [24], and improve sleep [22], physical activity and diet [25]. Parental support/involvement has been found to increase adolescent physical activity [26], while parental supply of alcohol, favourable attitudes, and monitoring are identified as important predictors of adolescent alcohol use [27]. Interventions delivered to parents afford the opportunity to target these modifiable parenting factors. For families experiencing socio-economic disadvantage, parent-based programs can target unique barriers, such as financial and household stress, and lack of social support [28], while eHealth (digital) interventions can improve accessibility. Parent-based interventions delivered alongside school-based programs can improve adolescent health behaviours [29,30]. Yet there are very few digital interventions that target multiple adolescent risk behaviours concurrently for parents experiencing socio-economic disadvantage [31]. To address this gap, the present study employs an innovative approach to co-design an effective, efficient eHealth intervention for parents experiencing socio-economic disadvantage, to accompany the existing *Health4Life* school-based program.

The Multiphase Optimisation Strategy (MOST) [32] is an engineering-inspired approach for developing, optimising, and evaluating interventions. Although RCTs are the gold standard for evaluating efficacy, they typically evaluate interventions as a whole, and do not provide information about which component or combination of components are most effective [32]. The MOST framework has the potential to promote the rapid, systematic development of preventive interventions by offering a fast, efficient way to test multiple intervention components simultaneously, and build interventions containing only demonstrably effective components [33]. MOST consists of three phases. In the *preparation phase*, a conceptual model is developed and intervention components are selected and tested. In the *optimisation phase*, a highly efficient experiment is conducted to test the individual and combined performance of the components. Results are used to guide assembly of an 'optimised intervention package' that achieves the greatest improvement in the target outcome, with the least resource consumption or burden. In the *evaluation phase*, an RCT is run to provide high-quality evidence about the efficacy of the optimised intervention. The MOST framework has been used to build multicomponent interventions for a range of public health issues [33,34] but has not yet been used to optimise a digital intervention to support parents to concurrently improve multiple adolescent health behaviours.

The preparation phase of MOST for the present study was conducted in 2024. First, we developed a conceptual model (Fig. 1) to guide the selection of intervention components, which was informed by principles from Social Cognitive Theory and Family Systems Theory, and findings from a survey with Australian parents [35] and a review of parent-based interventions [31]. Subsequently, we selected five intervention components: online learning modules, tailored feedback, stress management, text messages and health coaching. The online modules were included as a 'core component', to be delivered to all parents to provide them with essential content to improve their knowledge about the Big 6

health behaviours and enhance parenting self-efficacy, while the other four components were selected to be experimentally tested. Next, we partnered with parents and teenagers experiencing socio-economic disadvantage to iteratively co-design and user test the *Health4Life Parents and Teens* intervention. Full details of the preparation phase will be reported elsewhere. This paper focuses on the optimisation phase, and an evaluation phase is planned following completion of this study.

This paper describes the protocol for a factorial trial to optimise a digital intervention to support parents to improve multiple adolescent health behaviours. Specifically, this study aims to:

i) Test the individual and synergistic effects of four intervention components (tailored feedback, stress management, text messages and health coaching) for improving parental encouragement of adolescent health behaviours among parents experiencing socio-economic disadvantage over a 3-month period, and;
ii) Assemble an optimised intervention in which all components are effective and present the least possible burden to parents.

1. Methods

1.1. Design

A 2^4 (2x2x2x2) factorial design (i.e. four components with two levels each - on/off) will be implemented among parents experiencing socio-economic disadvantage in New South Wales, Australia in 2025. Parents will be randomised to one of 16 intervention combinations (see Table 1). Ethics approval was gained from the University of Sydney (2024/HE001483) and the University of Newcastle (R-2025-0006). Permission was also gained from the State Education Research Applications Process (SERAP 2024249) to promote the study via NSW Department of Education schools. This protocol follows the SPIRIT guidelines [36].

1.2. Study setting

This study will be conducted entirely remotely, via the study website (https://parentsandteens.org.au), SMS, and phone or video calls. Recruitment will be led by researchers at the University of Sydney and the University of Newcastle.

1.3. Eligibility criteria

Participants will be eligible if they are a parent/caregiver of an 11–15-year-old and currently living in a disadvantaged area of NSW (quintiles 1 or 2 of the Australian Bureau of Statistics' Index of Relative Socio-economic Disadvantage). If a parent has more than one 11–15-year-old, they will be instructed to complete the self-report questionnaires in relation to one child only for the duration of the study. To avoid contamination effects, only one parent per household will be eligible. Exclusion criteria include being unable/unwilling to provide consent, having no access to the internet at home or another suitable location or being identified as a 'bot' or disingenuous respondent.

Table 1
Experimental conditions in 2^4 factorial design.

Condition	Online Learning Modules *(Core Component)*	Tailored Feedback	Stress Management	Text Messages	Health coaching
1	**On**	**On**	**On**	**On**	**On**
2	**On**	**On**	**On**	**On**	Off
3	**On**	**On**	**On**	Off	**On**
4	**On**	**On**	**On**	Off	Off
5	**On**	**On**	Off	**On**	**On**
6	**On**	**On**	Off	**On**	Off
7	**On**	**On**	Off	Off	**On**
8	**On**	**On**	Off	Off	Off
9	**On**	Off	**On**	**On**	**On**
10	**On**	Off	**On**	**On**	Off
11	**On**	Off	**On**	Off	**On**
12	**On**	Off	**On**	Off	Off
13	**On**	Off	Off	**On**	**On**
14	**On**	Off	Off	**On**	Off
15	**On**	Off	Off	Off	**On**
16	**On**	Off	Off	Off	Off

On = receives component; Off = does not receive component.

1.4. Recruitment

Parents will be recruited via multiple channels, including social media, secondary schools, local newspapers, public transport, community organisations, and Local Health Districts (e.g. hospitals and community health centres). Data quality checks will be employed to manage any potential risk to recruitment presented by bots, as well as to avoid collection of data from disingenuous individuals [37]. To maximise recruitment, snowballing techniques will be used. Eligible participants will receive a $15 gift card if they successfully refer another eligible parent.

1.5. Participant timeline

Table 2 provides an overview of the schedule of events. Advertisements will provide parents with a weblink/QR code directing them to an eligibility screener. Eligible parents will be re-directed to an information statement to provide informed consent. Translated versions will be available, to cater for parents whose primary language is other than English. Consenting participants will be asked to complete a 20–30-min

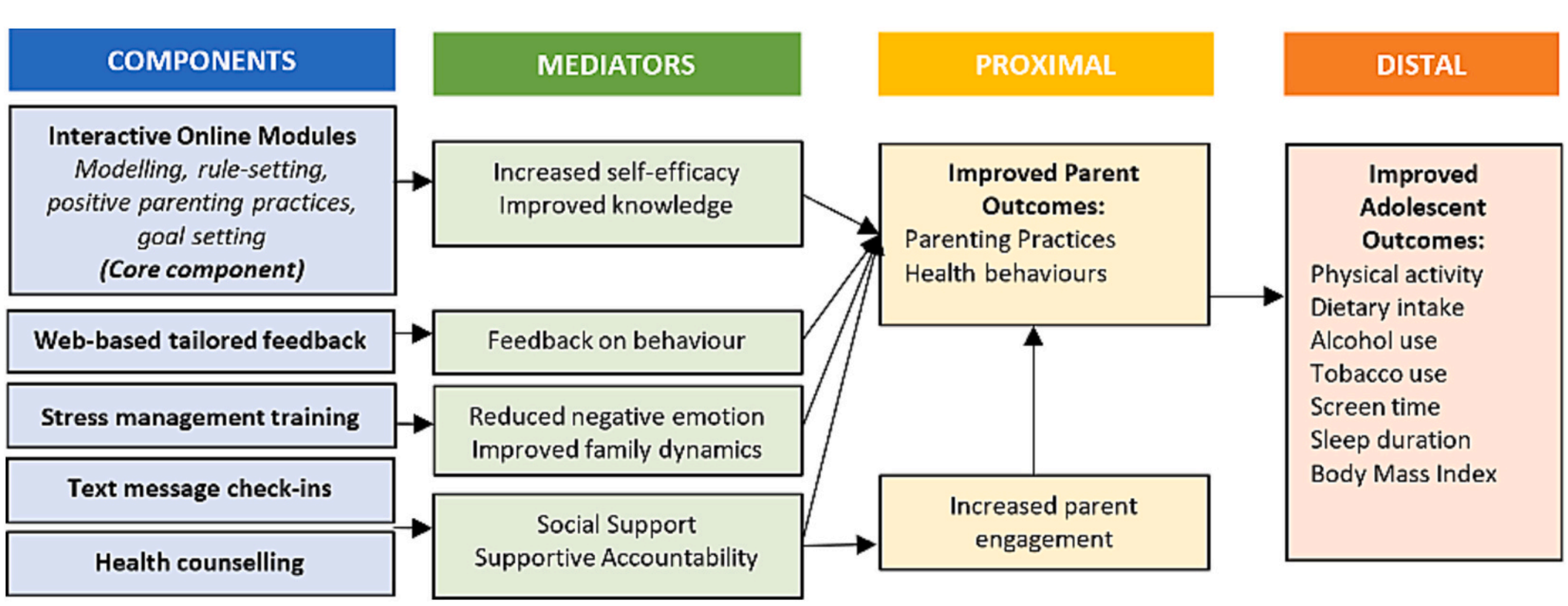

Fig. 1. Conceptual model.

Table 2
SPIRIT schedule of enrolment, interventions, and assessments.

	Study Period			
	Enrolment and baseline	Allocation	Intervention period	3-month follow-up
**Timepoint**	$-t_1$	**0**	t_1	t_2
**ENROLMENT**				
Eligibility Screening	X			
Informed consent	X			
Allocation		X		
**INTERVENTIONS**				
Health4Life Parents & Teens Online learning modules			◆————————◆	
Text Messages			X	
Health Coaching			X	
Stress Management Training			X	
Tailored Feedback			X	
**ASSESSMENTS**				
Demographics	X			X
Parental Encouragement of Health Habits[68]	X			X
Parent Health Behaviours[50-57]	X			X
Parental Self-Efficacy[58]	X			X
Health Literacy[59]	X			X
Parental Stress[61]	X			X
Parent-Adolescent Communication[64]	X			X
Supportive Accountability[65]	X			X
Process Outcomes[66]	X			X

online baseline survey. Next, participants will be randomised to one of the 16 experimental conditions, and subsequently emailed information on how to access the *Health4Life Parents & Teens* program and their allocated components. At the end of the 6–7-week intervention period, parents will be invited to complete an online evaluation to provide feedback about the program. Parents will also be sent an automated email invitation with a link to complete a 3-month follow-up survey. If participants do not complete the follow-up survey, email, SMS and phone call reminders will be made. Parents will be reimbursed a $30 e-gift voucher for completing each online survey.

1.6. Randomisation and blinding

A biostatistician with no role in recruitment will produce the randomisation scheme using the Blockrand function in R. Randomisation will proceed using a blocked random allocation method with randomly selected block sizes to ensure an equal number of participants in each group, while preventing selection bias that can occur when using fixed block sizes. Participants will be automatically randomised based on the previously determined randomisation scheme via the online REDCap system once they complete the baseline assessment. As is commonly the case for health intervention trials, it is not possible to blind participants to the nature of the intervention they are receiving. Similarly, research personnel need to be unblinded to provide access to correct components, however as all assessments will be conducted online, researchers will not be required to administer assessments. The study's statistician will be blinded to allocation when completing the analysis.

1.7. Sample size calculations

A previous RCT of a comparable web-based intervention among Australian parents found a small effect ($d = 0.27$) when examining pre-post intervention change on the Parenting to Reduce Adolescent Depression Anxiety Scale (PRADAS) [38]. However, even small effects can have large population health impact if the intervention is implemented broadly [39]. To achieve 80 % power to detect an effect size of 0.27 in our primary outcome (i.e. health habits PRADAS sub-scale), we estimated that we need 261 parent participants at the 3-month follow-up. To obtain an equal number of participants in each condition, we will need 272 participants (i.e. 17 per condition). To account for ~30 % attrition at 3-months, we will aim to recruit 389 participants.

1.8. Withdrawal

Participants can withdraw at any time without having to provide reason. The withdrawal process will be outlined in the participant

information statement and will include contact details for the chief investigator. If requested, withdrawal will include the removal of all participant-related research data, up until the point that data is analysed and published.

1.9. The Health4Life Parents & Teens intervention

The *Health4Life Parents & Teens* intervention comprises six online learning modules ('core intervention'), which will be delivered to all parents, regardless of randomisation. Four additional components were selected for testing: tailored feedback, stress management, text messages and health coaching. The components are designed to target modifiable parenting factors (mediators), to improve parenting practices, parent's own health behaviours and promote parent engagement with the intervention (proximal outcomes), and ultimately, to improve adolescent health behaviours (distal outcomes; Fig. 1). For the current trial, we chose to use a proximal outcome (parental encouragement of adolescent health habits) as our primary outcome, rather than the distal outcome of adolescent risk behaviours, which is an acceptable approach in MOST trials [32]. Importantly, adolescent outcomes will be assessed in the subsequent evaluation phase of MOST via an RCT with parent-adolescent dyads.

1.9.1. Online learning modules (core component)

All parents will receive access to the core intervention via the study website (www.parentsandteens.org.au), which comprises six modules, one module per risk factor: screen time and social media, smoking/vaping, alcohol, sleep, food and nutrition, and physical activity. The self-directed modules aim to empower parents to support their adolescent child to adopt healthy behaviours by enhancing parental self-efficacy and improving knowledge. Each module comprises a podcast-style video (~12 mins) with a health professional, followed by an interactive summary of parenting strategies, and goal setting. Content focuses on promoting healthy role-modelling, fair and consistent rule-setting, parental monitoring, and open communication (see Table 3). A strengths-based approach was adopted, with a focus on recognising and building upon existing family strengths and fostering positive behaviour change without shame or judgement. Established behaviour change theories underpin the content, including social learning theory, self-determination theory and ecological theory. Each module takes approximately 20-min to complete and is designed to be completed once per week, over a 6-week period. Access to subsequent modules will become available 7 days after the first module has been started. Parents will be provided with links to additional resources, such as how to promote healthy behaviours within financial constraints, and how to access services (e.g. food, housing).

1.9.2. Additional components

Participants will be randomly assigned to receive four intervention components with two levels (off/on).

1) Tailored feedback. Parents randomised to this component will receive tailored feedback about their parenting practices in relation to the "Big 6" risk behaviours via the study website, prior to completing their weekly online learning module. Each week, parents will be prompted to answer a one-item parenting-related question (aligning with the online module they are about to begin, e.g. "sleep"). The self-report questions were derived from our primary outcome [27]. Based on their responses (never, rarely, sometimes, often), parents will receive colour-coded (green, orange or red), strengths-based feedback and motivational advice about their encouragement of their teenager's health habits.
2) Stress management module. Parents randomised to this condition will have access to an additional online learning module about managing stress. Content will be delivered via a 10-min video/podcast featuring a registered psychologist, followed by an interactive

Table 3
Overview of core Health4Life Parents & Teens module content.

Module	Key messages
Screen time & social media	• Improving parental knowledge about adolescent screen time and social media use:<br>- Prevalence of screen use and Australian guidelines<br>- Negative effects of excessive screen time<br>- Impacts of engagement with different types of content online<br>• Strategies for parents to manage screen time:<br>- Setting rules and limits<br>- Role modelling good screen time habits (e.g. avoiding being on phone during family mealtimes)<br>- Parental monitoring (e.g. being aware of what your teenager is doing online)<br>- Managing resistance and push-back
Smoking and vaping	• Improving knowledge about adolescent tobacco smoking and e-cigarette use (vaping):<br>- Current rates of smoking and vaping among Australian teenagers<br>- Overview of vaping and their health risks<br>- Why early discussions on smoking and vaping matter<br>• Strategies for parents to help teenagers to avoid smoking/vaping:<br>- Setting clear rules and expectations<br>- Role modelling healthy habits (e.g. not smoking/vaping yourself)<br>- Educating teenagers about the harms and risks of smoking and vaping<br>- Supporting teenager to develop assertiveness and refusal skills<br>• Support for parents if their teenager is already smoking and/or vaping<br>- Guidance on how to help a teenager quit<br>- Access to resources (e.g., quit smoking services, GPs, counsellors)<br>- Mental health support options
Alcohol use	• Improving knowledge about adolescent alcohol use:<br>- Australian Alcohol Guidelines<br>- Patterns, prevalence, and harms related to alcohol use among Australian adolescents<br>- Benefits associated with not using alcohol or delaying onset of use<br>- Why teenagers may choose to/not to drink alcohol<br>• Strategies for parents to support their teenager to avoid drinking alcohol:<br>- Avoiding supply of alcohol to underage children/teenagers<br>- Setting rules and expectations (e.g. 'no drinking under 18 years old')<br>- Monitoring exposure to alcohol (e.g. being aware of their whereabouts and who they are with, and access to alcohol in the home)<br>- Role modelling healthy drinking habits<br>• Supporting teenager to develop assertive communication and refusal skills
Sleep	• Improving parental knowledge about adolescent sleep:<br>- The Australian 24-h movement guidelines<br>- Sleep patterns of Australian adolescents<br>- Importance of sleep for wellbeing and development in adolescence<br>- Factors affecting sleep for adolescents<br>• Parenting strategies to support optimal sleep among teenagers:<br>- Role modelling health sleepy habits (e.g., consistent bedtimes, wind down routines)<br>- Rule setting around healthy sleep habits with teenagers (e.g. consistent bed and wake times, limit active use of technology nearing bedtime)<br>• Creating a supportive home environment (e.g. evening wind down routines)
Physical activity	• Improving parental knowledge about adolescent physical activity:<br>- The Australian 24-h movement guidelines<br>- Short- and long-term benefits of being physically active (physical, mental, social)<br>• Strategies for parents to promote physical activity:

(continued on next page)

Table 3 (*continued*)

Module	Key messages
	- Role modelling<br>- Creating a supportive environment (e.g. provide ways to/from sport)<br>- Setting expectations around physical activity<br>- Helping teenagers find ways to be physically active that they enjoy
Food and nutrition	• Improving knowledge about adolescent food and nutrition:<br>- Australian dietary guidelines<br>- Over availability of 'discretionary' foods (e.g., ultra-processed food) and sugar sweetened beverages (SSB)<br>• Importance of increasing diet quality and variety. Parenting strategies to support healthy eating habits:<br>- Getting enough food on the table (including budget-friendly strategies), encouraging regular meal patterns<br>- Involving teenagers in meal planning and preparation<br>- Role modelling healthy eating habits and positive body image behaviours<br>- Eating mindfully, avoiding eating mindlessly (e.g. in front of screens)

summary of strategies, and goal setting. The key content covers the physical, mental, and social impacts of consistent stress, work-life balance, identifying and responding to stress triggers, managing financial stress, and self-care.

3) Text messages. Parents randomised to this condition will receive text messages to provide social support, supportive accountability and encourage intervention engagement. Based on previous research [40,41], a bank of text messages was developed in consultation with parents. Text messages will be semi-personalised (e.g. name) and automated via our study website, containing hyperlinks to prompt interaction with the website. Text messages will be triggered by interaction events with the core intervention (e.g. ready to begin a module, starting a module, completing a module). Participants will receive 2–3 text messages per week, staggered throughout a 7-day period.
4) Health coaching. Parents randomised to this component will receive weekly coaching calls with a trained health coach (minimum undergraduate training in health promotion, psychology or related field). Parents will have the option to request a translator when booking to ensure that English proficiency is not a barrier. Parents will choose to receive the coaching calls via phone or video call and will pick a suitable day and time. The health coach will follow a semi-structured manual which covers checking-in on progress with the intervention, troubleshooting technical issues or barriers to intervention engagement, and goal setting. Calls will be recorded, with a random subsample (10 %) to be scored by the research team to monitor fidelity with the protocol.

1.10. Data management

All participants will complete the online baseline and follow-up assessments via the secure Research Electronic Data Capture (REDCap) program. Access will be restricted to investigators named on the project. Data will be stored in a password protected database within the University-licenced Research Data Store platform, a secure, enterprise-grade Network Attached Storage device. The lead investigator of the project will be in full control of the dataset and permissions given to project members. Only members of the research team will be granted access to the stored data.

1.11. Outcome measures

1.11.1. Demographics

Parent age, sex at birth, gender identity, language/s spoken at home, country of birth, religion, postcode, marital status, number and age of children, employment, household income and highest level of education will be assessed. Participants will also be asked whether they or a family member has ever been diagnosed with prevalent chronic conditions e.g. heart disease or cancer. Food insecurity will be measured by a single item [42]. Demographic variables were selected to align with the PROGRESS-Plus framework, which promotes the collection of comprehensive, equity-relevant data [43,44].

1.11.2. Primary outcome

The Health Habits sub-scale of the Parenting to Reduce Adolescent Depression Anxiety Scale (PRADAS) [38]) will be used to measure parents' encouragement of their teenager's health habits. The sub-scale includes 12-items, such as *"I encourage [my teenager] to eat a healthy, balanced diet, including plenty of fresh vegetables and water"*. Parents will be asked to respond on a four-point scale ranging from "never" to "often". The PRADAS has been validated with Australian parents and adolescents [38]. Reliability was high for the total score, and convergent validity was supported by moderate to high correlations with two established measures of parenting. The health habits sub-scale scale was shown to have good internal consistency, high test-retest reliability, and significant convergent validity [38]. The full PRADAS questionnaire includes nine sub-scales which comprehensively assess a range of parenting risk and protective factors shown to influence the development of depression and anxiety disorders in adolescents. However, for the present study we chose only to include the health habits sub-scale.

1.11.3. Additional outcomes

Additional outcomes will be assessed at baseline and 3-month follow-up, as possible mediators and moderators of the efficacy of the individual intervention components.

1.11.3.1. Parent health behaviours. *Moderate-to-vigorous physical activity:* two items will be asked to calculate MVPA (minutes per week). *Screen time:* a single item will assess recreational (non-work) screen time (hours/day). *Dietary intake*: assessed via four items, which have been used in other large health behaviour change RCTs [15,45]. Examples of average portion sizes will be given. Serves/day of fruit and vegetable intake will be assessed via two similar items. Weekly sugar-sweetened beverage and sometimes food (i.e., ultra-processed food) intake will also be assessed. *Sleep*: Sleep duration (hours/day) will be calculated as the difference between wake time and bedtime. *Alcohol use:* quantity and frequency of consuming alcohol (standard drink) in the past 6 months. *Smoking/Vaping:* current smoking and vaping status, frequency of using cigarettes/vapes in the past 6 months. These measures have been used previously with parents [35] and are derived from validated scales [46–53].

1.11.3.2. Self-efficacy. Parental self-efficacy (a parent's belief in their ability to perform the parenting role successfully) will be measured using 10 items from the Efficacy subscale of the Parenting Sense of Competence Scale–Revised (PSOC-R) [54]. Responses will be made on a 5-point Likert scale ranging from "strongly disagree" to "strongly agree". The PSOC-R Efficacy subscale has been shown to be reliable and valid [54].

1.11.3.3. Health literacy. Parent's health literacy will be assessed using the HLS-EU-Q12, an adapted version of the European Health Literacy Survey Questionnaire (HLS-EU-Q47) [55]. Parents will respond to statements including, *"How easy would you say it is to find information on health attitudes such as exercise, healthy food and nutrition"*. Responses range from "very easy" to "very difficult". The HLS-EU-Q12 has demonstrated reliability and validity in adult populations [56].

1.11.3.4. Parental stress. Perceived parental stress will be assessed via the Parental Stress Scale (PSS), an 18-item questionnaire assessing parents' feelings about their parenting role, exploring both positive

aspects (e.g. emotional benefits, personal development) and negative aspects of parenthood (e.g. demands on resources, feelings of stress) [57]. Responses will be made on a scale from "strongly agree" to "strongly disagree". This measure has shown good internal consistency [57–59] and convergent validity [58].

1.11.3.5. Parent-adolescent communication. The Parent-Adolescent Communication Scale (PACS) will be used [60]. The instrument comprises two subscales (open communication and communication problems, 10-items each). Parents will respond to items on a 5-point Likert scale ranging from "strongly disagree" to "strongly agree". The PACS has shown good internal consistency, reliability and construct validity [60].

1.11.3.6. Supportive accountability. A modified version of the Accountability subscale (10-items) from the Supportive Accountability Measure will be used [61]. The scale aims to assess supportive accountability within the context of technology-mediated programs for adults. Participants are asked to rate support received from family, friends, and their health coach using a 7-point Likert scale ranging from "Not at all" to "Very much".

1.11.4. Process outcomes

Following completion of each module, parents will be asked to rate their overall experience (out of 5 stars) via the study website. In addition, a brief self-report questionnaire will be delivered to parents after the intervention period. Parents will be asked to rate the acceptability of the program as a whole, and of each component they received, on a 5-point scale. Parents will also be asked to rate the suitability of the length of each component, as well as the relevance, helpfulness, and ease of completion. Parents will also be asked to provide free-text responses in relation to any changes they would make to the program and specific things they liked about the modules.

To assess our secondary objective of assembling the most effective intervention that imposes the least possible burden on parents, parents will be asked to report the effort it took to complete each component they were assigned to ('no effort at all' to 'huge effort'). This item was adapted from an existing Theoretical Framework of Acceptability questionnaire [62]. Objective data will be collected via the study website to assess parents' engagement with each component (e.g., number of modules accessed, number completed, and the number of goals set). Health coaches will record the number of sessions attended, mode of communication, and the duration of each contact (see Supplementary Materials).

1.12. Analysis

Linear mixed effect (multilevel) models will test whether each intervention component had a minimum effect of $\geq d = 0.27$ on the primary outcome at 3-months, accounting for repeated measures nested within participants. Data will be analysed on an intent-to-treat basis and each intervention component will be coded using mean effect coding to enable interpretation of the regression coefficients (main effects) as the mean difference from the grand mean of all components. Random effects will be entered for each participant whereas time and component levels (on/off) will be entered as fixed effects. An interaction between time and component will be included to investigate significant change in the outcome over time. Next, models will test 2- and 3-way interactions between components to identify synergistic or antagonistic effects on the primary outcome. Results will be used to determine which components should be eliminated, and of those that remain, which set of components produces the best improvement on the primary outcome. We will use parent-reported perceived effort associated with completing each component to achieve our secondary aim of assembling an optimised intervention producing the greatest improvement in our primary outcome attainable, with the least possible burden to parents.

1.13. Data monitoring

The trial and any adverse events will be monitored by the research personnel and reviewed regularly by the Investigator team. The Trial Coordinator will be responsible for monitoring and reporting progress against milestones and deliverables at regular meetings. The investigators will permit study-related monitoring, audits, ethical review, and regulatory inspections. Any modifications to the study protocol will be recorded and submitted to the University of Sydney's HREC for approval.

1.14. Dissemination of results

Data will be analysed and published in peer-reviewed journals and presented at scientific conferences. A summary of results will be available to participants at the conclusion of the study. All data will be in aggregate form; no identifiable participant information will be published or presented.

2. Discussion

This paper describes the protocol of the *Health4Life* Parents and Teens Optimisation trial, the first study to use the novel MOST framework to build an effective and efficient digital parent-based intervention to improve the health behaviours of adolescents experiencing socio-economic disadvantage. A key strength is the use of the MOST framework to systematically and efficiently test the efficacy of each intervention component, allowing for elimination of more burdensome and less impactful components. This will yield an optimised, resource-efficient parent-based intervention to improve parental encouragement of the "Big 6" health risk behaviours among adolescents experiencing socio-economic disadvantage. Another key strength is the co-designed nature of the intervention, which included iterative collaboration with community members from the target group, to ensure the intervention is community-driven and relevant to Australian parents experiencing socio-economic disadvantage.

Possible limitations include reduced generalisability to a wider population of parents given our focus on parents experiencing socio-economic disadvantage from one Australian state only. However, we have chosen to focus on NSW as it is the most populous Australian state with racially, ethnically and socioeconomically diverse populations [63], which may better reflect the general Australian population. Second, we have specifically focused our measures on parent self-report, and do not include adolescent outcomes in this trial, which may be considered a limitation. This is the recommended approach within the 'optimisation phase' of the MOST framework, during which researchers can optimise the intervention based on mediators or proximal outcomes (i.e. impact of the parental intervention on changes in parental behaviour), rather than the ultimate (distal) outcome of changes in adolescent behaviours. Importantly, we will assess adolescent-reported health risk behaviours in the next phase of this study (evaluation phase), via a fully powered RCT.

3. Conclusion

This study adopts an innovative approach to develop a co-designed, scalable digital intervention to improve parental encouragement of adolescent health risk behaviours for families experiencing socioeconomic disadvantage. This will ultimately reduce inequitable health outcomes of a priority population of young Australians. Following this optimisation trial, the next step will be to evaluate the optimised *Health4Life* Parents and Teens program alongside the existing *Health4-Life* school-based program, in a new sample of parents and adolescents via a cluster RCT during the evaluation phase of MOST.

CRediT authorship contribution statement

Katrina E. Champion: Writing – original draft, Supervision, Methodology, Funding acquisition, Conceptualization. **Lily Davidson:** Writing – review & editing, Supervision, Project administration, Methodology. **Emily Hunter:** Writing – review & editing, Methodology. **Louise Thornton:** Writing – review & editing, Methodology, Funding acquisition. **Bonnie Spring:** Writing – review & editing, Methodology, Funding acquisition. **Bridie Osman:** Writing – review & editing, Writing – original draft, Methodology. **Matthew Sunderland:** Writing – review & editing, Methodology, Funding acquisition. **Cath Chapman:** Writing – review & editing, Funding acquisition. **Tracy Burrows:** Writing – review & editing, Methodology, Funding acquisition. **Tim Slade:** Writing – review & editing, Methodology, Funding acquisition. **Stephanie R. Partridge:** Writing – review & editing, Methodology, Funding acquisition. **Lauren Gardner:** Writing – review & editing, Funding acquisition. **Belinda Parmenter:** Writing – review & editing, Methodology, Funding acquisition. **Louise A. Baur:** Writing – review & editing, Methodology, Funding acquisition. **Maree Teesson:** Writing – review & editing, Funding acquisition. **Cathrine Mihalopoulos:** Writing – review & editing, Funding acquisition. **Alex Haidinger:** Writing – review & editing, Methodology. **Tara Finn:** Writing – review & editing, Methodology. **Lyra Egan:** Writing – review & editing, Funding acquisition. **George Johnson:** Writing – review & editing, Methodology, Funding acquisition. **Emily Stockings:** Writing – review & editing, Methodology, Funding acquisition. **Susan Woolfenden:** Writing – review & editing, Methodology, Funding acquisition. **Nicola C. Newton:** Writing – review & editing, Methodology, Funding acquisition. **David R. Lubans:** Writing – review & editing, Supervision, Methodology, Funding acquisition.

Funding sources

This study is funded by the Australian Government Department of Health's Medical Research Future Fund (MRF2021535). KC is funded by a University of Sydney Horizon Fellowship. LT is funded by a grant from the Medical Research Future Fund (MRF2017307). BS is funded by U.S. National Institutes of Health R01 grant DK125404. TB (#1173681), CC (#2026552), LAB (#2009035), LG (#2033054), MT (#1195284) and ES (#2017346) are funded by NHMRC Investigator Grants. SRP is funded by a Sydney Horizon Fellowship and a National Heart Foundation Future Leader Fellowship (#106646). GJ is funded by an In-Kind contribution from the SIWCF. The funders had no role in study design, writing of the paper, or the decision to submit the paper for publication.

Declaration of competing interest

The authors declare that they have no known competing financial interests or personal relationships that could have appeared to influence the work reported in this paper.

Acknowledgements

The authors would like to acknowledge the parents, adolescents and schools who participated in the co-design and testing of the *Health4Life Parents & Teens* program.

Appendix A. Supplementary data

Supplementary data to this article can be found online at https://doi.org/10.1016/j.cct.2025.107958.

Data availability

Upon completion of the study, data will be made available upon reasonable request to the study team.

References

[1] Organisation, W.H, Adolescent Health, Available from: https://www.who.int/health-topics/adolescent-health#tab=tab_1, 12 May 2025.

[2] R.M. Viner, et al., Adolescence and the social determinants of health, Lancet 379 (9826) (2012) 1641–1652.

[3] C. Wright, et al., Young adult cancer risk behaviours originate in adolescence: a longitudinal analysis using ALSPAC, a UK birth cohort study, BMC Cancer 21 (1) (2021) 365.

[4] K.E. Champion, et al., Lifestyle risks for chronic disease among Australian adolescents: a cross-sectional survey, Med. J. Aust. 216 (3) (2021) 156–157.

[5] K.D. Hesketh, et al., Results from the Australian 2022 report card on physical activity for children and young people, J. Exerc. Sci. Fit. 21 (1) (2023) 83–87.

[6] AIHW, Australia's Youth: In Brief, AIHW, Canberra, 2021.

[7] Australian Institute of Health and Welfare, Australia's Children. Cat. no. CWS 69, AIHW, Canberra, 2020.

[8] J.E. Kaikkonen, et al., Does childhood nutrition influence adult cardiovascular disease risk? Insights from the Young Finns Study, Ann. Med. 45 (2) (2013) 120–128.

[9] S.R. Partridge, et al., Addressing disparities: A systematic review of digital health equity for adolescent obesity prevention and management interventions, Obes. Rev. 25 (12) (2024) e13821.

[10] J.J. Prochaska, J.O. Prochaska, A review of multiple health behavior change interventions for primary prevention, Am. J. Lifestyle Med. 5 (3) (2011), https://doi.org/10.1177/1559827610391883.

[11] Partridge, S.R., et al., An analysis of current obesity strategies for adolescents in NSW against best practice recommendations: implications for researchers, policymakers and practitioners. Health Promot. J. Austr.

[12] K.E. Champion, et al., Effectiveness of school-based eHealth interventions to prevent multiple lifestyle risk behaviours among adolescents: a systematic review and meta-analysis, Lancet Digital Health 1 (5) (2019) e206–e221.

[13] J.J. Prochaska, B. Spring, C.R. Nigg, Multiple health behavior change research: an introduction and overview, Prev. Med. 46 (3) (2008) 181–188.

[14] K.E. Champion, et al., Health4Life eHealth intervention to modify multiple lifestyle risk behaviours among adolescent students in Australia: a cluster-randomised controlled trial, Lancet Digital Health 5 (5) (2023) e276–e287.

[15] S. O'Dean, S. Smout, M. Sunderland, T. Slade, L.A. Gardner, C. Chapman, L. Thornton, B. Osman, E. Hunter, L. Egan, M.T. Teesson, N.C. Newton, K. E. Champion, Adolescent Behavioural Intentions: Secondary Outcomes from a Cluster Randomised Controlled Trial of the Health4Life School-based Lifestyle Modification Intervention, Canadian Journal of Public Health, 2024 (In press).

[16] S. Smout, et al., Anxiety, depression and distress outcomes from the Health4Life intervention for adolescent mental health: a cluster-randomized controlled trial, Nat. Mental Health 2 (7) (2024) 818–827.

[17] L. Egan, et al., Process evaluation of the digital Health4Life intervention among a sample of disadvantaged adolescents and teachers, Health Promot. Int. 39 (6) (2024) daae170.

[18] C. Wade, A. Seward, A. Almendingen, E. Robinson, Parenting Pre-teens: A Pivotal Time for Children and Parents, 2019.

[19] M.R. Weiss, A.C. Phillips, Motivation in youth sport and physical activity: developmental perspectives, in: J.D. Wright (Ed.), International Encyclopedia of the Social & Behavioral Sciences (Second Edition), Elsevier, Oxford, 2015, pp. 914–920.

[20] J. Povey, et al., Adolescence a period of vulnerability and risk for adverse outcomes across the life course: The role of parent engagement in learning, in: J. Baxter, et al. (Eds.), Family Dynamics over the Life Course: Foundations, Turning Points and Outcomes, Springer International Publishing, Cham, 2022, pp. 97–131.

[21] T. Sanvictores, M.D. Mendez, Types of parenting styles and effects on children, in: StatPearls, StatPearls Publishing, 2025.

[22] S.P.H. Khor, et al., Modifiable parental factors in adolescent sleep: A systematic review and meta-analysis, Sleep Med. Rev. 56 (2021) 101408.

[23] T.A. Barnett, et al., Sedentary behaviors in today's youth: approaches to the prevention and Management of Childhood Obesity: A scientific statement from the American Heart Association, Circulation 138 (11) (2018) e142–e159.

[24] J. Leonardi-Bee, M.L. Jere, J. Britton, Exposure to parental and sibling smoking and the risk of smoking uptake in childhood and adolescence: a systematic review and meta-analysis, Thorax 66 (10) (2011) 847.

[25] M. Verloigne, et al., Family- and school-based correlates of energy balance-related behaviours in 10–12-year-old children: a systematic review within the ENERGY (EuropeaN energy balance research to prevent excessive weight gain among youth) project, Public Health Nutr. 15 (8) (2012) 1380–1395.

[26] H.E. Brown, et al., Family-based interventions to increase physical activity in children: a systematic review, meta-analysis and realist synthesis, Obes. Rev. 17 (4) (2016) 345–360.

[27] M.B.H. Yap, et al., Modifiable parenting factors associated with adolescent alcohol misuse: a systematic review and meta-analysis of longitudinal studies, Addiction 112 (7) (2017) 1142–1162.

[28] J.M. Jurkowski, et al., The empowerment of low-income parents engaged in a childhood obesity intervention, Fam. Community Health 37 (2) (2014) 104–118.

[29] K.E. Champion, et al., Parent-based interventions to improve multiple lifestyle risk behaviors among adolescents: A systematic review and meta-analysis, Prev. Med. 164 (2022) 107247.

[30] L.K. Chai, et al., Effectiveness of family-based weight management interventions for children with overweight and obesity: an umbrella review, JBI Database System Rev. Implement. Rep. 17 (7) (2019) 1341–1427.

[31] K.E.A. Champion, A systematic review and meta-analysis of parent-based interventions to improve multiple lifestyle risk behaviours among adolescents and parents, in: Australasian Society for Behavioural Health and Medicine, 2022. Virtual.
[32] L.M. Collins, Optimization of Behavioral, Biobehavioral, and Biomedical Interventions: The Multiphase Optimization Strategy (MOST), Springer, Pittsburgh, 2018.
[33] B. Spring, et al., A factorial experiment to optimize remotely delivered behavioral treatment for obesity: results of the opt-IN study, Obesity 28 (9) (2020) 1652–1662.
[34] M.E. Piper, et al., Toward precision smoking cessation treatment I: moderator results from a factorial experiment, Drug Alcohol Depend. 171 (2017) 59–65.
[35] K.E. Champion, et al., Parental information needs and intervention preferences for preventing multiple lifestyle risk behaviors among adolescents: cross-sectional survey among parents, JMIR Pediatr. Parent 6 (2023) e42272.
[36] SPIRIT, SPIRIT Statement: Standard Protocol Items: Recommendations for Interventional Trials, January 10, 2025]; Available from: https://spirit-statement.org/, 2025.
[37] J. Comachio, et al., Identifying and counteracting fraudulent responses in online recruitment for health research: a scoping review, BMJ Evid. Based Med. 30 (2024) 173–182, https://doi.org/10.1136/bmjebm-2024-113170.
[38] M.C. Cardamone-Breen, et al., The parenting to reduce adolescent depression and anxiety scale: assessing parental concordance with parenting guidelines for the prevention of adolescent depression and anxiety disorders, PeerJ 5 (2017) e3825.
[39] E.C. Matthay, et al., Powering population health research: considerations for plausible and actionable effect sizes, SSM Popul. Health 14 (2021) 100789.
[40] L.K. Chai, et al., Development of text messages targeting healthy eating for children in the context of parenting partnerships, Nutr. Diet. 76 (5) (2019) 515–520.
[41] S.R. Partridge, et al., Not to be harsh but try less to relate to 'the teens' and you'll relate to them more': co-designing obesity prevention text messages with adolescents, Int. J. Environ. Res. Public Health 16 (24) (2019).
[42] Statistics, A.B.O, Australian Health Survey: Nutrition First Results - Foods and Nutrients, ABS, 2011.
[43] J. O'Neill, et al., Applying an equity lens to interventions: using PROGRESS ensures consideration of socially stratifying factors to illuminate inequities in health, J. Clin. Epidemiol. 67 (1) (2014) 56–64.
[44] Group, C.E.M, PROGRESS-Plus Framework, Available from: https://methods.cochrane.org/equity/projects/evidence-equity/progress-plus, 2025.
[45] M. Teesson, et al., Study protocol of the Health4Life initiative: a cluster randomised controlled trial of an eHealth school-based program targeting multiple lifestyle risk behaviours among young Australians, BMJ Open 10 (7) (2020) e035662.
[46] F.K. Del Boca, J. Darkes, The validity of self-reports of alcohol consumption: state of the science and challenges for research, Addiction 98 (s2) (2003) 1–12.
[47] L. Harrison, et al., Comparing Drug Testing and Self-Report of Drug Use among Youths and Young Adults in the General Population, 2007.
[48] K. Larsson, et al., Criterion validity and test-retest reliability of SED-GIH, a single item question for assessment of daily sitting time, BMC Public Health 19 (1) (2019) 17.
[49] B. Nguyen, D. Ding, S. Mihrshahi, Fruit and vegetable consumption and psychological distress: cross-sectional and longitudinal analyses based on a large Australian sample, BMJ Open 7 (3) (2017) e014201.
[50] N.D. Ridgers, et al., Physical activity during school recess: a systematic review, Am. J. Prev. Med. 43 (3) (2012) 320–328.
[51] I.H.E. Rutishauser, K. Webb, B. Abraham, R. Allsopp, Evaluation of Short Dietary Questions from the 1995 National Nutrition Survey, National Food and Nutrition Monitoring and Surveillance Project, Commonwealth Department of Health and Aged Care, 2001.
[52] M.A. Short, et al., Estimating adolescent sleep patterns: parent reports versus adolescent self-report surveys, sleep diaries, and actigraphy, Nat. Sci. Sleep 5 (2013) 23–26.
[53] Health, A.I.O. and Welfare, National Drug Strategy Household Survey 2019, AIHW, Canberra, 2020.
[54] L. Gilmore, M. Cuskelly, The parenting sense of competence scale: updating a classic, Child Care Health Dev. 50 (1) (2024) e13173.
[55] K. Sørensen, et al., Measuring health literacy in populations: illuminating the design and development process of the European health literacy survey questionnaire (HLS-EU-Q), BMC Public Health 13 (1) (2013) 948.
[56] J.M. Pelikan, et al., Measuring comprehensive, general health literacy in the general adult population: the development and validation of the HLS19-Q12 instrument in seventeen countries, Int. J. Environ. Res. Public Health 19 (2022), https://doi.org/10.3390/ijerph192114129.
[57] J.O. Berry, W.H. Jones, The parental stress scale: initial psychometric evidence, J. Soc. Pers. Relat. 12 (3) (1995) 463–472.
[58] J.J. Zelman, M.A. Ferro, The parental stress scale: psychometric properties in families of children with chronic health conditions, Fam. Relat. 67 (2) (2018) 240–252.
[59] S. Algarvio, I. Leal, J. Maroco, Parental stress scale: validation study with a Portuguese population of parents of children from 3 to 10 years old, J. Child Health Care 22 (4) (2018) 563–576.
[60] H.L. Barnes, D.H. Olson, Parent-adolescent communication and the circumplex model, Child Dev. 56 (2) (1985) 438–447.
[61] K. Chhabria, et al., The assessment of supportive accountability in adults seeking obesity treatment: psychometric validation study, J. Med. Internet Res. 22 (7) (2020) e17967.
[62] M. Sekhon, M. Cartwright, J.J. Francis, Development of a theory-informed questionnaire to assess the acceptability of healthcare interventions, BMC Health Serv. Res. 22 (1) (2022) 279.
[63] Statistics, A.B.O, National, State and Territory Population, Available from, https://www.abs.gov.au/statistics/people/population/national-state-and-territory-population/latest-release, 2024.
