# Supplementary material for: Optimising a digital parent-based intervention to improve adolescent health risk behaviours in families living in socio-economically disadvantaged communities: findings from the Health4Life Parents & Teens factorial randomised trial
Source: Lancet Reg Health West Pac. 2026 Sep 23;74:101987. doi: 10.1016/j.lanwpc.2026.101987 (PMC13649520; doi:10.1016/j.lanwpc.2026.101987)
Supplement: Supplementary file 2 [file mmc1.docx]

**Optimising a digital parent-based intervention to improve adolescent health risk behaviours in families experiencing socio-economic disadvantage: Findings from the Health4Life Parents & Teens factorial randomised trial**

**Supplementary Materials**

[1.	Example search strategy for systematic review of parent-based intervention	2](#_Toc240628120)

[2.	Conceptual model for Health4Life Parents & Teens	4](#_Toc240628121)

[3.	Data Quality Checking Protocol	5](#_Toc240628122)

[4.	Intervention components – Example screenshots	6](#_Toc240628123)

[5.	Summary of demographic measures included in the current study	11](#_Toc240628124)

[6.	Results from parsimonious regression model	14](#_Toc240628125)

### 1. **Example search strategy for systematic review of parent-based intervention**

**Database: Ovid MEDLINE(R) ALL <1946 to May 05, 2021>**

**Search Strategy:**

1 (parent*3 or guardian* or mum or mom or mother* or father* or dad or grandparent* or grandmother* or grandfather* or famil*).tw. (1741824)
2 Parents/ or Parenting/ or Legal Guardians/ or Mothers/ or Fathers/ or Grandparents/ or Family/ (201172)
3 1 or 2 (1788899)
4 (adolescen* or child* or teen* or minor* or juvenile* or pubescen* or prepubescen* or kid* or youth*).tw. (2461832)
5 Adolescent/ or Child/ or Minors/ (2891948)
6 4 or 5 (4257335)
7 (alcohol* or intoxicat* or underage drink*).tw. (385482)
8 ((alcohol or drink*) adj2 (abus* or us* or disorder* or consum* or heav* or problem* or excess* or bing* or risk* or reckless*)).tw. (126490)
9 Alcohol Drinking/ or Alcoholic Intoxication/ or Binge Drinking/ or Underage Drinking/ or Alcoholism/ (142847)
10 7 or 8 or 9 (424486)
11 (smok* or tobacco or cigarette* or vaping or vape or ecig* or e-cig*).tw. (353709)
12 Smoking/ or Tobacco Smoking/ or Cigarette Smoking/ or Tobacco Products/ or Vaping/ or Electronic Nicotine Delivery Systems/ (150806)
13 11 or 12 (383096)
14 (physical* activ* or exercis* or sport* or physical* exert* or play* or danc* or recreation* or fitness or aerobic* or run* or walk* or jump* or energ* or swim* or gym*).tw. (2798156)
15 Exercise/ or High Intensity Interval Training/ or exp Sports/ or Physical Exertion/ or "Play and Playthings"/ or Dancing/ or Recreation/ or Physical Fitness/ or exp Running/ or exp Walking/ or Swimming/ or Gymnastics/ (363730)
16 14 or 15 (2897249)
17 (diet or food or nutrition or feeding behavio* or eat* or meal* or lunch or breakfast or dinner or supper or snack* or appetite or overeat* or undereat* or overnutrit* or undernutrit* or malnutrit* or underweight or overweight or obes* or overnourish* or undernourish* or malnourish* or fast food* or weight).tw. (1889646)
18 ((over or under) adj1 (eat* or nutrit* or weight or nourish*)).tw. (6120)
19 Diet/ or Diet, Healthy/ or Energy Intake/ or Food/ or Feeding Behavior/ or Eating/ or exp Meals/ or Appetite/ or Malnutrition/ or Thinness/ or Overweight/ or Obesity/ or Obesity, Abdominal/ or Obesity, Morbid/ or Pediatric Obesity/ or Fast Foods/ or Body Weight/ (686651)
20 17 or 18 or 19 (2086796)
21 (inactiv* or sedentary or sit*).tw. (2338282)
22 (screen* adj1 (time* or recreation* or activit*)).tw. (5335)
23 Sedentary Behavior/ or Sitting Position/ or Screen Time/ (11665)
24 21 or 22 or 23 (2344339)
25 (sleep* or insomnia*).tw. (203881)
26 Sleep/ or Sleep Deprivation/ or Sleep Hygiene/ or "Sleep Initiation and Maintenance Disorders"/ (73931)
27 25 or 26 (215801)
28 (10 and 13) or (10 and 16) or (10 and 20) or (10 and 24) or (10 and 27) or (13 and 16) or (13 and 20) or (13 and 24) or (13 and 27) or (16 and 20) or (16 and 24) or (16 and 27) or (20 and 24) or (20 and 27) or (24 and 27) (1041210)
29 (intervention* or prevent* or program* or education* or health literacy or health promotion).tw. (3437279)
30 Internet-Based Intervention/ or Program Evaluation/ or Patient Education as Topic/ or Health Education/ or Consumer Health Information/ or Health Literacy/ or Health Promotion/ (272428)
31 29 or 30 (3543544)
32 randomized controlled trial.pt. (529170)
33 controlled clinical trial.pt. (94146)
34 (randomized or randomised).ab. (620475)
35 placebo.ab. (217665)
36 Clinical Trials as Topic/ (195693)
37 randomly.ab. (356731)
38 trial.ti. (239385)
39 32 or 33 or 34 or 35 or 36 or 37 or 38 (1403705)
40 exp Animals/ not Humans/ (4821824)
41 39 not 40 (1294214)
42 3 and 6 and 28 and 31 and 41 (2964)
43 limit 42 to yr="2010 -Current" (342)

### 2. **Conceptual model for Health4Life Parents & Teens**

### 3. **Data Quality Checking Protocol**

Participants were flagged as ‘potentially fraudulent’ if they met any of the below criteria:

- Eligibility screener, consent form or baseline survey completed between 12am and 6am (AEST/AEDT).
- Participant provided an email address that includes multiple numbers (e.g., [LouiseT4560@gmail.com](mailto:LouiseT4560@gmail.com)). The experience of other experienced digital health researchers suggests that emails with a long number at the end are often provided by disingenuous participants.
- If they provided a phone number outside of Australia (i.e., not starting with +61) or outside of NSW (i.e., not beginning with 02) or that did not fit the format of standard phone numbers for Australian landlines (8 digits plus area code) or mobile phone numbers (10 digits plus country code).
- Contact information provided by a participant (e.g. email or phone number) matched information provided by a previous participant.
- The participant did not provide a valid/eligible response to which State they live in (via a ‘free text’ response to a new question in the eligibility survey which asked: “Which State do you live in?”)
- IP address attached to their online survey response indicated they were located outside of Australia.
- “Are you currently using a Virtual Private Network (VPN) on this computer/phone?” (yes/no/unsure) item was used to assess whether IP addresses that appeared as being outside of Australia could have been caused by the participant using a VPN.
- Time taken to complete eligibility survey was under the minimum threshold set (i.e. response was done so quickly it is unlikely to have been completed by a real person) and/or multiple responses that start/end at the same time
- Respondent answered ‘honeypot’ question (immediate exclusion if response recorded – bot only)
- Respondent incorrectly answered “What is your favourite local restaurant or local grocery store near where you live?” question
- Respondent did not select ‘Agree’ for attention check question

All eligible participants, regardless of whether they were flagged as potentially fraudulent, received an identity verification phone call, as indicated in the eligibility screener (“*Please note: If you are eligible for the study, we will contact you briefly via phone call to confirm your identity before you are enrolled in the program and sent your gift card”)*. This procedure was implemented both as a verification step and as a deterrent to fraudulent participation. Participants who were not flagged on any fraud indicators were included in the study irrespective of whether their identity could be successfully verified within a maximum of two contact attempts. In contrast, participants who were flagged on one or more of the above fraud criteria were only included if their identity could be successfully verified within two contact attempts (e.g., via direct contact or an appropriate voicemail confirming identity). Participants whose identity could not be verified after two attempts were excluded from the study and notified of this outcome via email.

### 4. **Intervention components – Example screenshots**

**Online Learning Modules**

**Tailored Feedback**

**Text Messages**

### 5. **Summary of demographic measures included in the current study**

| **Outcome** | **Detail** |
| --- | --- |
| Age | **What is your age?** |
| Sex at birth | **What sex were you assigned at birth? (i.e. the one that was originally specified on your birth certificate)**   - Male - Female - Prefer not to say |
| Gender Identity | **How do you describe your gender? (Please select the most appropriate response)**   - Man - Woman - Non-binary/ gender fluid - Prefer not to answer - I use a different term (please specify): _________________________ |
| Language/s spoken | **Do you speak any languages other than English at home?**   - Yes (please specify: __________) - No |
| Country of birth | **In which country were you born?** **___________________________** |
| Highest level of education | **What is the highest level of education you have achieved (or equivalent qualification from your country of completion)?**   - Never attended school and no non-school qualification - Year 9 or below - Year 10 - Year 11 - Year 12 - Certificate I/II - Certificate III/IV - Advanced Diploma / Diploma - Bachelor degree - Graduate Diploma / Graduate Certificate - Postgraduate Degree - Certificate not further defined - Don’t know/Prefer not to say |
| Employment | **How would you best describe your employment situation at the moment?**   - Employed full time - Employed part time or casual - Unemployed - Student - Retired - Permanently unable to work - Home duties - Other: ___________________ |
| Medical conditions/diagnoses | **Have you ever been diagnosed with any of the following conditions?**   - Arthritis - Asthma - Cancer. *If yes, please select which cancer:* - Breast - Bowel (including colorectal, colon, rectum) - Prostate - Lung - Kidney - Pancreatic - Liver - Mouth (including oesophageal) - Other ________________________ - Heart Disease (I.e. cardiovascular disease, stroke, heart attack etc.) - High Blood Pressure - High Cholesterol - Kidney Disease - Osteoporosis or osteopenia - Type 1 Diabetes - Type 2 Diabetes - Chronic Obstructive Pulmonary Disease (COPD) - Non-alcoholic fatty liver disease (NAFLD) - No, none |
| Marital Status | **What is your marital status?**   - Single - Married - Divorced - De facto - Never married - Widowed - Separated - Prefer not to say |
| Household Income | **What is your annual household income?**   - Nil income-$18,200 - $$18,201-$45,000 - $45,001-$120,000 - $120,001-$180,000 - $180,001 and over - Prefer not to say |
| Food Security | **In the last 12 months was there any time you have run out of food and not been able to purchase more?** (McKay et al., 2019)   - Yes - No - Prefer not to say |
| Number and age of children | **How many children do you have? (drop-down list)**   - 0 - 1 - 2 - 3 - 4 - 5 - 6+ |
| Parental stress | The Parental Stress Scale (PSS; Berry & Jones, 1995) is an 18-item questionnaire assessing parents’ feelings about their parenting role, exploring both positive aspects (e.g. emotional benefits, personal development) and negative aspects of parenthood (e.g. demands on resources, feelings of stress). |

### 6. **Results from parsimonious regression model**

| **Term** | **Estimate** | **SE** | **t_value** | **p_value** |
| --- | --- | --- | --- | --- |
| **(Intercept)** | 5.49 | 0.61 | 9.04 | <0.0001 |
| **Time** | 0.52 | 0.11 | 4.74 | <0.0001 |
| **Feedback** | 0.09 | 0.10 | 0.88 | 0.38 |
| **Coaching** | -0.10 | 0.10 | -1.01 | 0.31 |
| **Stress** | -0.05 | 0.09 | -0.62 | 0.54 |
| **Text** | 0.03 | 0.09 | 0.34 | 0.74 |
| **Parent Age** | 0.03 | 0.01 | 2.22 | 0.03 |
| **Feedback:Stress** | 0.03 | 0.10 | 0.28 | 0.78 |
| **Feedback:Text** | -0.07 | 0.10 | -0.68 | 0.49 |
| **Feedback:Coaching** | -0.21 | 0.10 | -2.18 | 0.03 |
| **Coaching:Stress** | 0.06 | 0.10 | 0.57 | 0.57 |
| **Coaching:Text** | 0.03 | 0.10 | 0.27 | 0.79 |
| **Stress:Text** | 0.02 | 0.09 | 0.18 | 0.86 |
| **Time:Feedback** | 0.23 | 0.11 | 2.06 | **0.04** |
| **Time:Coaching** | 0.33 | 0.11 | 3.01 | **0.003** |
| **Feedback:Stress:Text** | 0.06 | 0.10 | 0.63 | 0.53 |
| **Feedback:Coaching:Stress** | 0.03 | 0.10 | 0.27 | 0.79 |
| **Feedback:Coaching:Text** | -0.10 | 0.10 | -1.03 | 0.31 |
| **Coaching:Stress:Text** | -0.05 | 0.10 | -0.51 | 0.61 |
| **Time:Feedback:Stress** | 0.05 | 0.11 | 0.43 | 0.67 |
| **Time:Feedback:Text** | 0.17 | 0.11 | 1.56 | 0.12 |
| **Time:Feedback:Coaching** | 0.13 | 0.11 | 1.15 | 0.25 |
| **Time:Coaching:Stress** | -0.17 | 0.11 | -1.58 | 0.11 |
| **Time:Coaching:Text** | -0.10 | 0.11 | -0.96 | 0.34 |
| **Feedback:Coaching:Stress:Text** | -0.16 | 0.10 | -1.67 | 0.10 |
| **Time:Feedback:Stress:Text** | 0.01 | 0.11 | 0.10 | 0.92 |
| **Time:Feedback:Coaching:Stress** | -0.01 | 0.11 | -0.09 | 0.93 |
| **Time:Feedback:Coaching:Text** | 0.15 | 0.11 | 1.37 | 0.17 |
| **Time:Coaching:Stress:Text** | 0.09 | 0.11 | 0.86 | 0.39 |
| **Time:Feedback:Coaching:Stress:Text** | 0.12 | 0.11 | 1.14 | 0.25 |
